# Cleaning the Cellular Factory–Deletion of McrA in *Aspergillus oryzae* NSAR1 and the Generation of a Novel Kojic Acid Deficient Strain for Cleaner Heterologous Production of Secondary Metabolites

**DOI:** 10.3389/ffunb.2021.632542

**Published:** 2021-02-09

**Authors:** Trong T. Dao, Kate M. J. de Mattos-Shipley, Ian M. Prosser, Katherine Williams, Marija K. Zacharova, Colin M. Lazarus, Christine L. Willis, Andrew M. Bailey

**Affiliations:** ^1^School of Chemistry, University of Bristol, Bristol, United Kingdom; ^2^School of Biological Sciences, University of Bristol, Bristol, United Kingdom

**Keywords:** heterologous host, *Aspergillus*, natural products, *mcrA*, secondary metabolism, kojic acid, NSAR1, NSARΔK

## Abstract

The use of filamentous fungi as cellular factories, where natural product pathways can be refactored and expressed in a host strain, continues to aid the field of natural product discovery. Much work has been done to develop host strains which are genetically tractable, and for which there are multiple selectable markers and controllable expression systems. To fully exploit these strains, it is beneficial to understand their natural metabolic capabilities, as such knowledge can rule out host metabolites from analysis of transgenic lines and highlight any potential interplay between endogenous and exogenous pathways. Additionally, once identified, the deletion of secondary metabolite pathways from host strains can simplify the detection and purification of heterologous compounds. To this end, secondary metabolite production in *Aspergillus oryzae* strain NSAR1 has been investigated via the deletion of the newly discovered negative regulator of secondary metabolism, *mcrA* (multicluster regulator A). In all ascomycetes previously studied *mcrA* deletion led to an increase in secondary metabolite production. Surprisingly, the only detectable phenotypic change in NSAR1 was a doubling in the yields of kojic acid, with no novel secondary metabolites produced. This supports the previous claim that secondary metabolite production has been repressed in *A. oryzae* and demonstrates that such repression is not McrA-mediated. Strain NSAR1 was then modified by employing CRISPR-Cas9 technology to disrupt the production of kojic acid, generating the novel strain NSARΔK, which combines the various beneficial traits of NSAR1 with a uniquely clean secondary metabolite background.

**Graphical Abstract:**
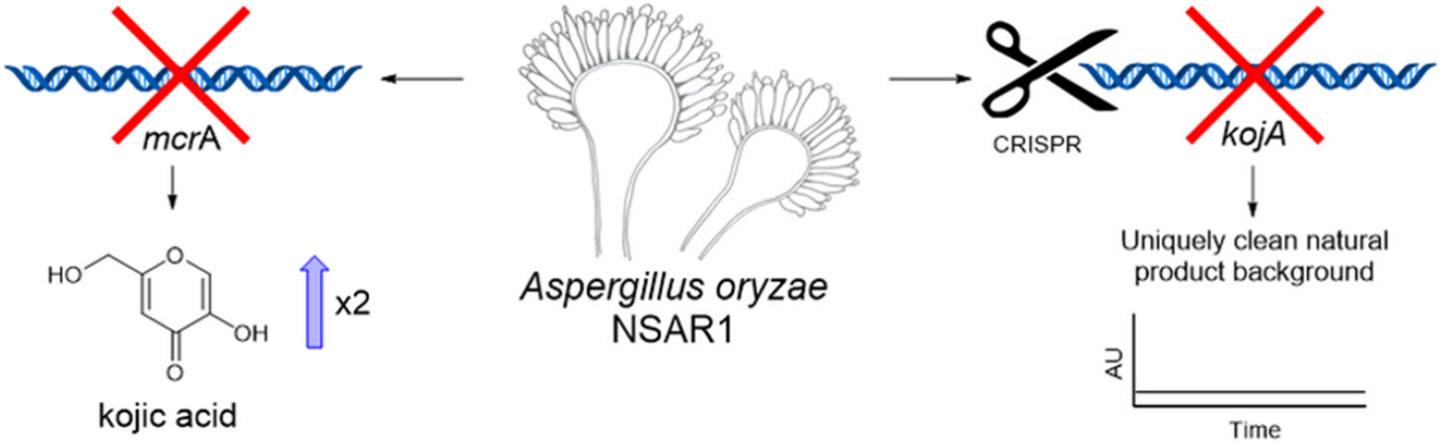
Deletion of *mcrA* led to an increase in kojic acid yields in *A. oryzae*. Disruption of kojic acid production using CRISPR-Cas9 generated a novel NSAR1-based strain with a uniquely clean background for the heterologous production of natural products.

## Introduction

Natural products are hugely valuable small molecules, often of microbial origin, which are used in many industries including food and cosmetics, and particularly in medicine due to their varied and potent bioactivities. A pressing need for new lead compounds, especially antibiotics (Theuretzbacher et al., 2019), is driving ongoing natural product discovery, especially in relatively under-exploited groups of microorganisms such as filamentous fungi. An essential approach in natural product research is the heterologous expression of secondary metabolite (SM) pathways. This technique can be employed as a tool to investigate SM biosynthesis, as a way of discovering novel compounds, and as a means of production. The filamentous ascomycete *Aspergillus oryzae* has been used extensively as a heterologous host for the production of secondary metabolites (Watanabe et al., 1998; Heneghan et al., 2010; Chiba et al., 2013; Williams et al., 2016). Various factors make this fungus a suitable platform for such work: it has GRAS (“Generally Recognized As Safe”) status due to its long history of use in food and drink production, is generally atoxic (Barbesgaard et al., 1992), and importantly, produces few natural products. Thus, *A. oryzae* provides a relatively clean background for the production of heterologous compounds.

Strain engineering to improve *A. oryzae* as a heterologous host has included the development of the multiply auxotrophic strain NSAR1, which can be transformed with up to four separate integrative plasmids using different selectable complementation markers (Jin et al., 2004). This greatly improves the capacity of *A. oryzae* to express complex natural products, which often require the expression of multiple pathway genes. This capacity has been further expanded by construction of a set of corresponding plasmids, each containing four different expression cassettes that provide strong levels of expression in *A. oryzae*. Complementation alone therefore allows for the co-expression of up to 16 different genes in the host organism, while deployment of similar plasmids based on dominant selectable markers (the *bar* gene for resistance to glufosinate ammonium and *ble* gene for resistance to phleomycin) could provide a theoretical total of 24 expressible transgenes (Pahirulzaman et al., 2012; Lazarus et al., 2014).

*A. oryzae* has been successfully used as a platform for the expression of fungal natural product pathways using the system described above (Wasil et al., 2013; Lazarus et al., 2014; Bailey et al., 2016; Williams et al., 2016), as well as in similar independently developed vector systems (Fujii et al., 2011; Tagami et al., 2013). Such work has allowed complex biosynthetic pathways to be studied in detail, contributing significantly to the understanding of fungal SM biosynthesis and uncovering novel chemistry. Engineered *A. oryzae* strains can also be used to generate novel and potentially valuable structural diversity, through pathway engineering and biotransformations. Alberti et al. (2017) recently demonstrated the value of such an approach, establishing *A. oryzae* as a platform for the production of semi-synthetic derivatives of the valuable antibiotic pleuromutilin, one of which demonstrated enhanced antibiotic activity. This was achieved through the bio-conversion of chemically modified intermediates that were fed to a transgenic *A. oryzae* strain.

**Figure 1:**
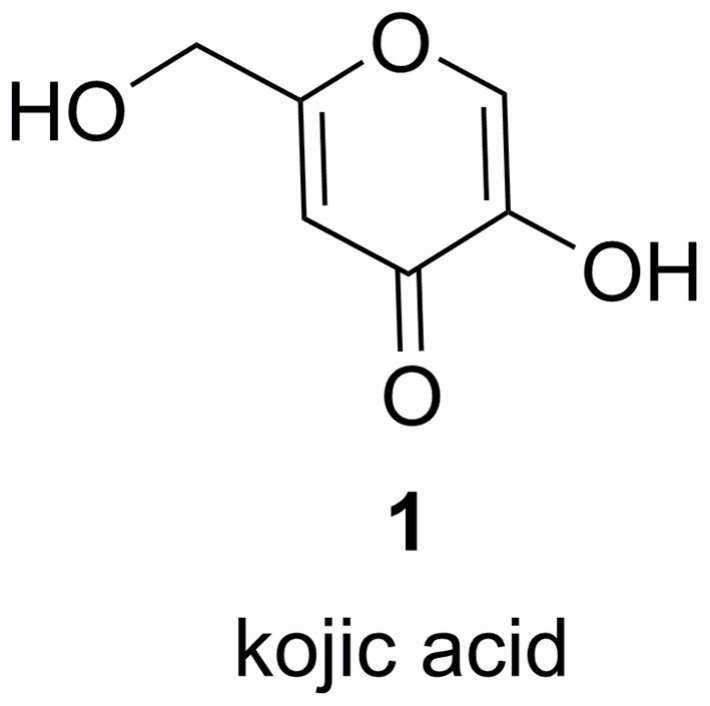
Structure of kojic acid **1**.

To fully exploit and further optimize NSAR1 as a “cellular factory,” it is important to characterize the natural metabolic capabilities of this strain. This would allow the exclusion of *A. oryzae* compounds when analyzing transformants and provide a better understanding of any potential interplay between endogenous *A. oryzae* metabolic pathways and the introduced natural product pathways. The main metabolite for which *A. oryzae* is known is kojic acid **1** (Figure 1), which is a sought-after product, believed to improve health and beauty, and used as a chelating agent in various industries including food and cosmetics (Bentley, 2006). Various strains of *A. oryzae* have been shown to produce additional secondary metabolites as detailed in a recent review by Frisvad et al. (2018), but in some cases these strains have been misidentified, and in general *A. oryzae* is known to be a poor producer of SMs.

The relatively low abundance of metabolites reported for *A. oryzae* strains is a characteristic that appears to have evolved since its divergence from the closely related *A. flavus*. *A. oryzae* is thought to be a “domesticated” relative of *A. flavus* (Barbesgaard et al., 1992); a hypothesis which is supported by genome analysis demonstrating that these species are 99.5% identical (Rokas et al., 2007). Comparison of gene expression profiles from SM biosynthetic gene clusters (BGCs) between *A. oryzae* and *A. flavus* shows that although numerous SM gene clusters are present in the genome of *A. oryzae*, they have been downregulated and, in many cases, appear totally silent under the tested conditions (Gibbons et al., 2012).

Recently, a novel negative regulator of secondary metabolism, McrA (multicluster regulator A), has been discovered in *A. nidulans*, where it has been shown to regulate at least 10 SM BGCs (Oakley et al., 2017). Deletion of *mcrA* was shown to stimulate SM production, even in strains carrying a deletion of the global SM regulator *laeA* (Bok and Keller, 2004). *McrA* appears to be conserved across many ascomycetes, with deletion of *mcrA* homologs in *A. terreus* and *Penicillium canescens* also resulting in increased SM production (Oakley et al., 2017).

The initial aim of this research was to identify and delete the *mcrA* homolog in *A. oryzae* in an attempt to induce SM production and thus better understand the metabolic capabilities of *A. oryzae* strain NSAR1. It was reasoned that the identification of functional biosynthetic pathways in *A. oryzae* could also direct strain improvements by targeting such pathways for deletion, thus creating an even cleaner background for future work employing *A. oryzae* as a cellular factory.

## Results

### Identification of an *A. oryzae mcrA* Homolog

The *A. oryzae mcrA* homolog was identified by searching for any *A. oryzae* protein sequences on the NCBI non-redundant protein database using the *A. nidulans* McrA protein sequence as the query [accession number AN8694 (Oakley et al., 2017)]. Three homologous putative proteins were identified from three separate *A. oryzae* strains; *A. oryzae* 3.042 (Zhao et al., 2012), *A. oryzae* BCC7051 (Thammarongtham et al., 2018), and *A. oryzae* RIB40 (Machida et al., 2005) (accession numbers: EIT82532.1, OOO05597.1 and XP_023092425.1, respectively). These three predicted proteins were all different lengths (366, 318, and 234 amino acids, respectively). The latter two appear to be truncated at the N-terminus and have an additional 22 amino acid sequence internally (Supplementary Figure 1). Interrogating the genome sequences of each strain and re-annotating each gene manually determined that these apparent differences were due to incorrect annotation. The full protein sequence previously annotated in the genome of strain 3.042 (Zhao et al., 2012) is present and conserved with 100% identity in every strain. This protein sequence shares 62% identity with the *A. nidulans* McrA with 98% query coverage (Supplementary Figure 2). This is equivalent to that found and reported for other *Aspergillus* strains (Oakley et al., 2017), suggesting that this protein is also conserved in *A. oryzae*. A GAL4-like Zn_2_Cys_6_ binuclear cluster DNA-binding domain (accession number cd00067) was identified from residues 118–157, supporting the previous assertion that McrA is likely to function as a transcription factor, or have a similar DNA binding function.

### Disruption of *Ao_mcrA*

To disrupt *Ao_mcrA*, a bipartite strategy (Nielsen et al., 2006) was used. *A. oryzae* NSAR1 was transformed with the relevant bi-partite knock-out fragments, containing homology to the *mcrA* flanking regions and the *argB* cassette for selection, and after multiple rounds of subculturing on selective media, nine of the resulting transformants were analyzed by PCR to evaluate integration of the targeting cassette and subsequent gene disruption (Supplementary Figure 3). Transformant *Ao*Δ*mcrA*-7 passed all diagnostic tests, with PCRs confirming correct integration of both bipartite fragments, and with the transformant being shown to be genetically pure by the absence of any wild-type *mcrA* (Supplementary Figure 3).

**Figure 2:**
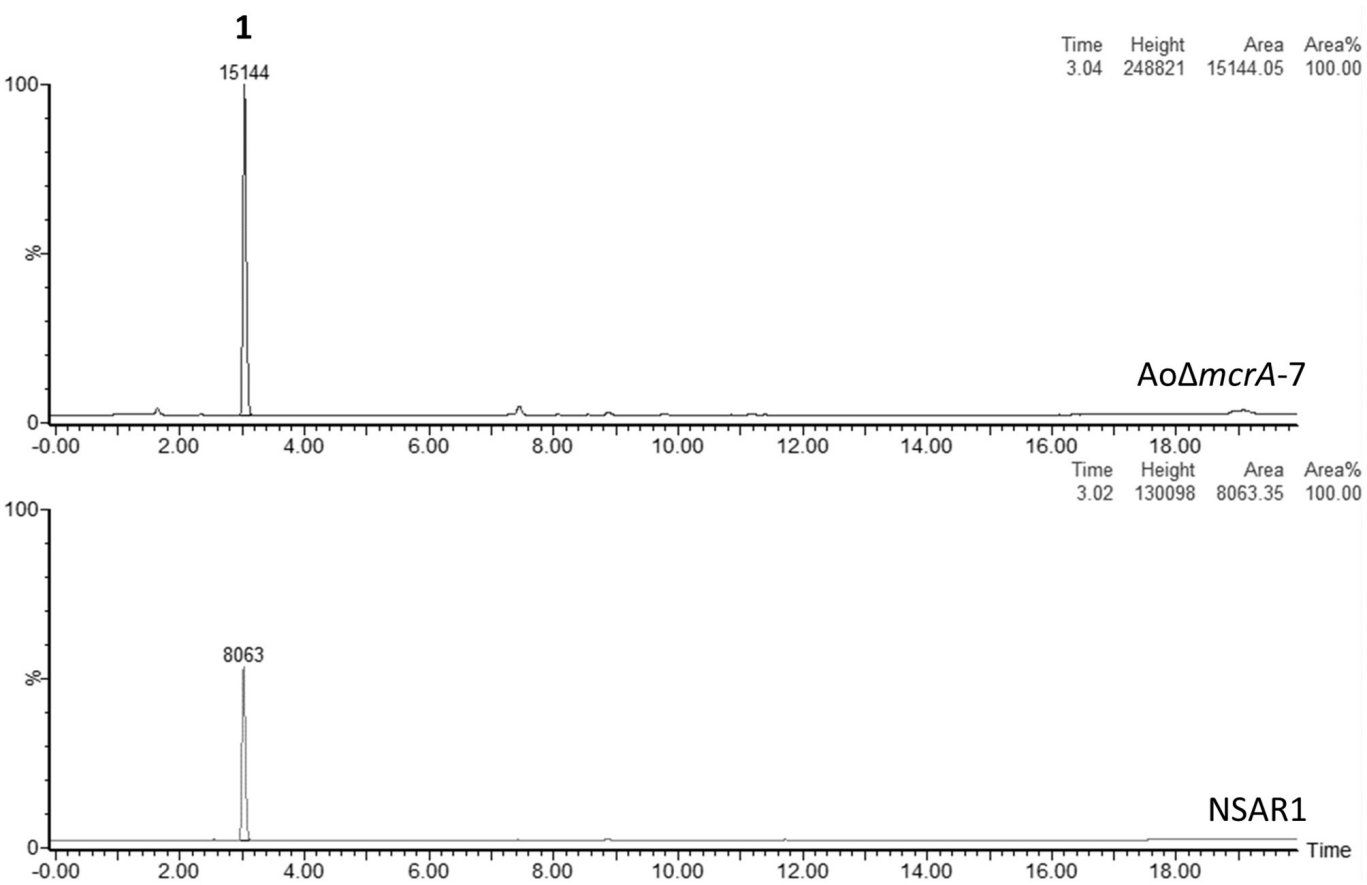
LCMS traces (ELSD) comparing the crude extracts of *A. oryzae* strain NSAR1 (bottom trace) and the *mcrA* deletion strain *Ao*Δ*mcrA*-7 (top trace). The only observable difference between the strains is an increase in kojic acid production (eluting at 3 min) in strain *Ao*Δ*mcrA*-7. The yields were quantified at 1.23 and 2.52 g/L for NSAR1 and *Ao*Δ*mcrA*-7, respectively.

Metabolite production in strain *Ao*Δ*mcrA*-7 was assessed on a range of media; namely CMP, MEB, PDP, GN, and static rice, and compared to the parental strain; *A. oryzae* NSAR1, to identify any changes in the metabolic profile as a result of the gene disruption. On all media, the metabolic profiles were found to be similar, with no novel metabolites observed. CMP cultures were then used for further analysis, including purification and quantification, as this is the medium routinely employed for heterologous production of compounds in *A. oryzae*, and supports high yields of crude extract. Both NSAR1 and *Ao*Δ*mcrA*-7 were fermented in CMP media for 1 week in triplicate. Quantitative analysis by LCMS again demonstrated that there were no dramatic changes in the metabolic profile of *Ao*Δ*mcrA*-7 (Figure 2), with the only evident change being an increase in the production of kojic acid from 1.23 to 2.52 g/L (Supplementary Table 1).

Fermentation of Δ*mcrA*-7 was scaled up to purify minor compounds for NMR analysis and subsequent structural elucidation (Supplementary Figures 4–42). In addition to kojic acid **1**, a kojic acid dimer **2** was also shown to be present in the extracts. Five diketopiperazines, **3**–**7**, were also isolated and characterized as the known *cyclo*-[(_D_)Pro-(_L_)Tyr] (**3**), *cyclo*-[(_D_)Pro-(_L_)Val] (**4**), *cyclo*-[(_D_)Pro-(_L_)Ile] (**5**), *cyclo*-[(_D_)Pro-(_L_)Leu] (**6**), and *cyclo*-[(_D_)Pro-(_L_)Phe] (**7**) (Figure 3). Some of these compounds have previously been reported as being isolated from *Aspergillus* species (Dong et al., 2014; Shaaban et al., 2014; Uchoa et al., 2017). However, a comparison to extractions from CMP medium containing no *A. oryzae* demonstrated that in this case, these compounds were likely derived from the growth medium and not *A. oryzae* (Supplementary Figure 43).

**Figure 3:**
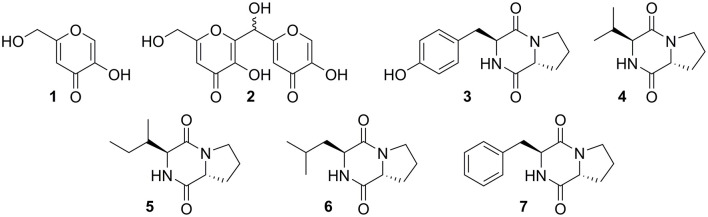
Compounds extracted from fermentation of Δ*mcrA*-7 in CMP medium.

### Generation of the *kojA* Mutant Strain NSARΔK

To disrupt kojic acid production in strain NSAR1 *kojA* was mutated using CRISPR-Cas9 technology. *KojA* encodes an FAD-dependent oxidoreductase shown previously to be necessary for kojic acid production (Terabayashi et al., 2010). A plasmid named pAYargCas9-kojA2 was constructed, containing Cas9 under the control of the inducible *P*_*amyB*_ promoter and a guide RNA cassette consisting of the Cas9-binding scaffold sequence and target-specific guide RNA, placed between the hammerhead (HH) and hepatitis delta virus (HDV) ribozymes. This design was based on that of Nødvig et al. (2015) and the use of ribozymes removes the requirement for an RNA polymerase III promoter. The targeting guide RNA (sequence GTCGCTCGAACAAAGAAACTCGG; complementary to nucleotides 90–109 of *kojA*) was designed using the online tool WU-CRISPR (Wong et al., 2015), and chosen based on its high predicted potency and 5′ location. An Interpro analysis (Mitchell et al., 2019) of KojA identified an FAD dependent oxidoreductase domain (IPR006076) located at residues 9–319, placing the target sequence toward the N-terminus of this domain. The approach used here is known to generate double-strand breaks (DSBs) at the target locus, with repair via the NHEJ pathway likely to introduce small indel (insertion-deletion) mutations (Nødvig et al., 2015). It was reasoned that any resulting indel mutations at this position, and particularly any out-of-frame mutations, would likely lead to a loss of function. pAYargCas9-kojA2 was also designed to contain the *AMA1* autonomously replicating sequence, to allow for the subsequent loss of the plasmid and recycling of the selectable marker.

**Figure 4:**
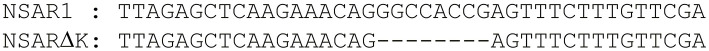
Sequencing of *kojA* in the deletion strain NSARΔK identified an 8 bp deletion.

Plasmid pAYargCas9-kojA2 was used to transform *A. oryzae* strain NSAR1, generating 42 transformants, which were transferred onto secondary plates lacking arginine. After two rounds of subculturing under such selection, transformants were transferred to KA-Fe medium. This medium contains iron trichloride (FeCl_3_), which reacts with kojic acid to produce a distinct red color, thus acting as an initial screen of kojic acid production. One transformant, *Ao*-ko18, failed to trigger such a color change and was taken forward for further analysis. A ~1 kb region covering the targeted *kojA* site was amplified from both the original NSAR1 strain and *Ao*-ko18 and sequenced using primer pair kojA-F/kojA-R. An alignment of the sequences identified an 8 bp deletion in the mutant strain precisely at the target site (Figure 4). This out-of-frame deletion is sufficient for a loss of function for *kojA*.

To fully confirm a loss of kojic acid production a chemical extraction was performed for *Ao*-ko18 fermentation cultures and the metabolic profile of the resulting crude extract was analyzed by both TLC and HPLC analysis (Supplementary Figures 44, 45). This confirmed *Ao*-ko18 as an essentially “empty” strain, producing no major compounds under our laboratory production conditions.

*Ao*-ko18 was then subcultured multiple times on non-selective medium (MEA), to allow for the loss of the autonomously replicating plasmid pAYargCas9-kojA2. This loss was confirmed by restoration of arginine auxotrophy, and the strain renamed *A. oryzae* NSARΔK.

**Figure 5:**
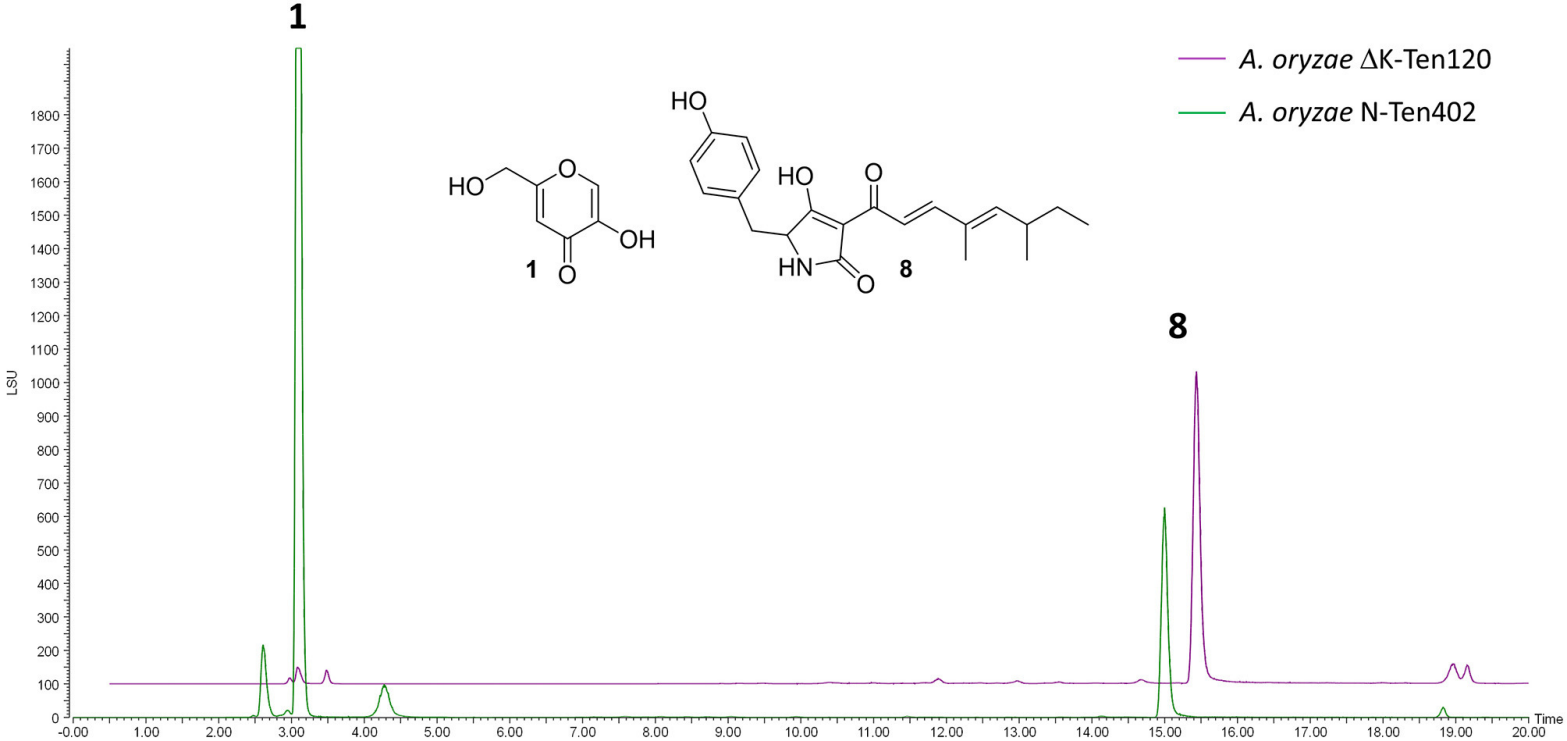
LCMS traces (ELSD) comparing CMP-agar crude extracts of *A. oryzae* strains heterologously producing pretenellin A **8** (eluting at 14.94 min). Strain N-Ten402 was generated by transforming NSAR1 and is therefore producing kojic acid **1** in addition to pretenellin A **8**. Strain ΔK-Ten120 was generated using NSARΔK and is producing almost exclusively pretenellin A **8**.

NSARΔK, and the original NSAR1 strain were both transformed with the pretenellin A **8** producing plasmid pTYGS-arg-TenC+TenS (Yang et al., 2019), to demonstrate the potential of using NSARΔK as a platform for the heterologous production of secondary metabolites. Two resulting strains, ΔK-Ten120 and N-Ten402, were fermented in production medium to induce the expression of the tenellin genes *TenS* and *TenC*. Both strains were confirmed as producing pretenellin A **8** in similar yields. In the case of ΔK-Ten120, the absence of kojic acid meant that the crude extract was almost pure pretenellin A **8** (Figure 5). The presence of kojic acid in large quantities can hinder the purification and downstream use of heterologously produced metabolites, making NSARΔK a valuable platform for the heterologous production of secondary metabolites.

## Discussion

A 366 amino acid protein – named Ao_mcrA – was identified as being encoded in the genomes of *Aspergillus oryzae* strains 3.042, BCC7051, and RIB40. This protein shares 62% protein identity with the characterized McrA of *Aspergillus nidulans* (Oakley et al., 2017), demonstrating a similar level of homology to that found and reported for McrA proteins from other *Aspergillus* species (Oakley et al., 2017). The conserved GAL4-like Zn2Cys6 binuclear cluster DNA-binding domain could also be detected within the predicted protein sequence of Ao_mcrA.

Unlike in filamentous fungi previously investigated, disruption of *Ao_mcrA* was shown to have little impact on secondary metabolite (SM) production in *A. oryzae* strain NSAR1, with the only observable change being an increase in the already major metabolite, kojic acid, from 1.23 to 2.52 g/L. As kojic acid is a valuable and commercially produced compound, a similar deletion of McrA could be conducted in any producing strains as a potentially straightforward way of increasing yields. The lack of impact the *Ao_mcrA* deletion had on the NSAR1 metabolome provides further evidence that SM production has been downregulated in *A. oryzae* when compared to ancestral *Aspergillus* species, and that this downregulation has occurred through means other than McrA-mediated repression. Previous studies have shown that both *A. oryzae* and the closely related *A. flavus* are actually enriched in genes for secondary metabolism when compared to other *Aspergillus* species (Payne et al., 2006), suggesting that *A. oryzae* does have the genetic potential for SM production, but where *A. flavus* is a prolific producer of secondary metabolites, gene clusters in *A. oryzae* are downregulated (Gibbons et al., 2012). One interesting avenue for further research would be to determine whether McrA impacts the transcriptome of *A. oryzae* and thus assess whether the expression of gene clusters has been decoupled from McrA regulation, or whether a factor other than transcription is impacting the SM profile of *A. oryzae*. It would also be intriguing to determine whether McrA-mediated repression of secondary metabolism functions as a key regulatory factor in *A. flavus*, and thus whether changes in the role of McrA have occurred during the recent domestication of *A. oryzae*.

Although kojic acid **1** is a commercially valuable metabolite, its production can be problematic in the context of using *A. oryzae* as a heterologous host, making chemical extractions and purification of metabolites more time-consuming and costly. Therefore, CRISPR-Cas9 technology was harnessed to halt kojic acid **1** production in an NSAR1 derived strain, through targeted mutation of *kojA*, an FAD-dependent oxidoreductase shown previously to be necessary for kojic acid production (Terabayashi et al., 2010). The plasmid construction strategy was intentionally designed so that the addition of the target-specific sgRNA cassette was the final step. This means that the intermediate plasmid, pAYargCas9, which contains both the Cas9 and AMA1 cassettes but not the sgRNA cassette, could easily be adapted to target other loci in strain NSAR1. Targeting *kojA* led to the generation of a strain containing an 8 bp deletion at the target site, with a resulting loss of function and subsequent inability to produce kojic acid. The *kojA* mutant strain, named NSARΔK, was then used as a host for the production of pretenellin A **8**, demonstrating the potential value of this strain as a platform for the production of commercially important heterologous compounds.

Slightly surprisingly, the CRISPR-Cas9 approach used here was found to be relatively inefficient, with only one out of 42 *A. oryzae* transformants demonstrating a loss of function for KojA. The low rate of disruption was not especially problematic in this instance, as rapid screening of kojic acid production is possible, but may be a hindrance in cases where disruption does not lead to a clear mutant phenotype. It is possible that the efficiency of targeting could be improved by the addition of a template for homology directed repair. Another consideration worth noting is the possibility of off-target mutations, which are known to occur with CRISPR-Cas9 editing (Zhang et al., 2015, Zischewski et al., 2017). Although multiple *A. oryzae* genome sequences are available, there is not currently a publicly available genome sequence for *A. oryzae* NSAR1. Sequencing the genomes of both NSAR1 and the novel strain NSARΔK would allow for an assessment of off-target mutations in strain NSARΔK and would provide genome data to support any further applications of these useful strains.

Finally, the isolation of apparent minor metabolites from crude extracts, which were later identified as diketopiperazines originating in the culturing medium, serves as a useful warning to the natural product community to consider all possible sources of small molecules and to document their presence for exclusion from future analyses.

Together these findings advanced our understanding of *A. oryzae* NSAR1 and led to the development of a new strain for heterologous production, namely strain NSARΔK. As a filamentous factory, *A. oryzae* NSARΔK offers a uniquely clean background.

## Materials and Methods

### Strains and Culture Conditions

*Escherichia coli* strain TOP10 (Invitrogen) was used as a host for plasmids and was maintained according to standard procedures (Sambrook and Russell, 2001). *Saccharomyces cerevisiae* strain YPH499 (Stratagene) was used for plasmid assembly by homologous recombination and was maintained on YPAD medium (1% (w/v) yeast extract, 2% (w/v) peptone, 2% (w/v) glucose, 0.004% (w/v) adenine sulfate, 1.5% (w/v) agar) at 28°C. *A. oryzae* NSAR1 was obtained as a gift from the Kitamoto group and was maintained on MEA medium (Sigma) at 28°C (Jin et al., 2004).

### Transformation of *A. oryzae*

Transformation of *A. oryzae* was performed according to the protocol outlined by Williams et al. (2016).

### *A. oryzae* Nucleic Acid Extraction

*A. oryzae* strains were cultured in 10 ml GN medium [2% (w/v) D(+)-glucose monohydrate, 1 % (w/v) Nutrient broth No. 2 (Oxoid, UK)] at 28°C with shaking at 200 rpm, then pelleted, lyophilised and ground under liquid nitrogen. Genomic DNA was prepared using the GenElute Plant Genomic DNA Miniprep kit (Sigma).

### Plasmid Construction for *Ao_mcrA* Deletion

*Ao_mcrA* was deleted from *A. oryzae* strain NSAR1 using the bipartite method (Nielsen et al., 2006). This method relies on the splitting of the selection marker (with a ~500 bp overlap), which leads to the requirement for homologous recombination between the two selection marker halves for selection to occur. This requirement for homologous recombination leads to higher gene disruption levels.

**Figure 6:**
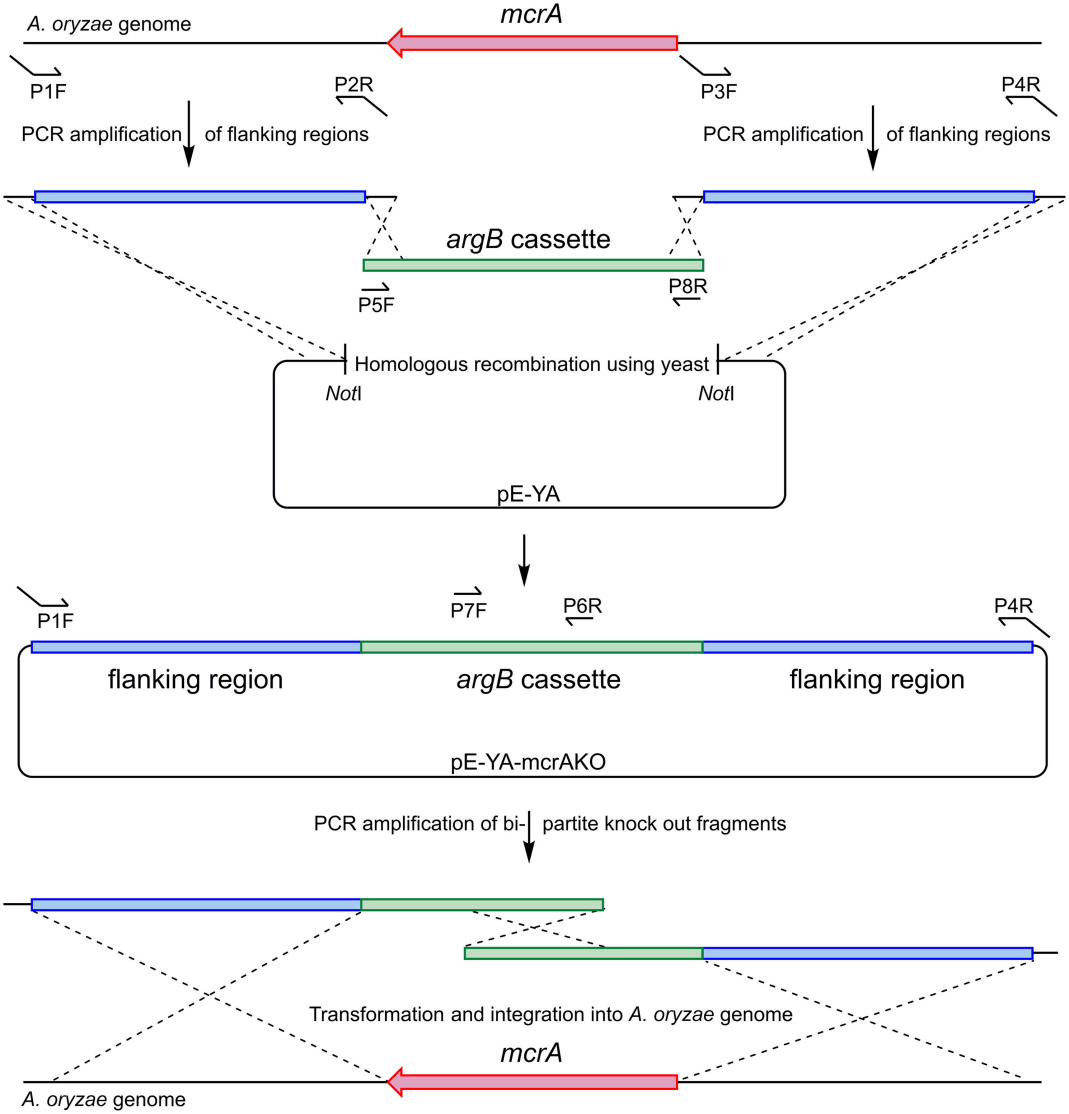
Construction of bi-partite fragments used for the deletion of *Ao_mcrA* and recombination into the *A. oryzae* genome.

A plasmid was constructed using homologous recombination in *S. cerevisiae* to provide a template for the bi-partite fragment amplification (Figure 6). Flanking regions either side of *Ao_mcrA* were amplified using the proof-reading polymerase KAPA HiFi HotStart (Roche), with the primer pairs AO-mcrAKO-P1F/AO-mcrAKO-P2R and AO-mcrAKO-P3F/AO-mcrAKOP4R. The *argB* cassette which provides selection in *A. oryzae* NSAR1 was amplified using primer pair PargB-P5F/TargB-P8R using the plasmid pTYarg as the template (Lazarus et al., 2014). All three fragments were recombined into the *E. coli*/yeast shuttle plasmid pE-YA (Pahirulzaman et al., 2012), which had been linearised with *Not*I. The resulting recombinant plasmid, pE-YA-mcrAKO, was used as a template for PCR amplification of the bi-partite fragments using primers AO-mcrAKO-P1F with argB-P6R, and argB-P7F with AO-mcrAKOP4R. Primers used are shown in Table 1.

**Table 1:** Primers used in this work. Compound primers, with tails (underlined) were designed to allow for yeast recombination.

**Primer name**	**Sequence 5^′^−3^′^**
AO-mcrAKO-P1F	ATGCCAACTTTGTACAAAAAAGCAGGCTCC CCTATGTCAACTCACGTTCG
AO-mcrAKO-P2R	TCAAATGAGGCCCAACTGATCTTCAGCATC CATATTCTCGATACGCCTGC
AO-mcrAKO-P3F	TGAACGAACCACTGCATCATCAGTCTAGAG CAGGATGATGTGAGAGTCG
AO-mcrAKO-P4R	AATGCCAACTTTGTACAAGAAAGCTGGGTC CCTTGAGATCTCCATCTGC
PargB-P5F	GATGCTGAAGATCAGTTGG
argB-P6R	ACCATCAAACTCCTTCAGC
argB-P7F	CAACTAGGTGTCAACGAGTC
TargB-P8R	CTCTAGACTGATGATGCAGTG
AO-mcrA-test-F	GTGTCTGTTTCTCTATCGTCC
AO-mcrA-test-R	GAATTGTCCGTGGTACCTC
pArgB-Bg-R	TCCTACCTCTCGCTTCATC
TargB-Nd-F	GCAAGAGCTCAACTCCTATTC
AO-mcrA-F	ATCCACCTCCTCAGTGGAAC
AO-mcrA-R	TGACTAGCATGCAGAGGAGG
PamyB-Cas9-F	CCCACAGCAAGCTCCGAATTGGATATC ATGGACAAGAAGTACTCCATTGG
Cas9-Asc-R	CGCCGCGACTTAGACAGGCGAGCCGAAAGG
Cas9-Asc-F	CGCCAAGGCTATCCTTTCGGCTCGCCTGTC
Cas9-TamyB-R	TCCACCCTTCACGAGCTACTACAGATC TCACACCTTCCTCTTCTTCTTGG
Pgpd-kojA2-HH-F	CAGCTACCCCGCTTGAGCAGACATCACCGTCGAGC CTGATGAGTCCGTGAGGACGAAACG
Guide-kojA2-R	GCTCTAAAACCCGAGTTTCTTTGTTCGAGCGAC GAGCTTACTCGTTTCGTCCTC
HH-kojA2-Guide-F	ACGAAACGAGTAAGCTCGTCGCTCGAACAAAGAAACTCGG GTTTTAGAGCTAGAAATAGC
Teno-HDV-R	CAGGTTGGCTGGTAGACGTCATATAATCATAC GTCCCATTCGCCATGCCG
kojA-F	ATTGGATACTGTCTTGCGGA
kojA-R	GTCCTCGGTGTGGATGATAC
AMA1-2F	CCCGATGGAGCTCTAGCTGG
AMA1-2R	AATCTCCTCGAGATGGCTGG
AMA1-1F	TTCTCATCTTTGACAGCTTATCATCGATAA GGCCAGTGCCAAGCTTAACG
AMA1-1R	TCTTCCTTCTGTTCGGAGATTACCGAATCA CAGCGGAAACAGCTATGACC

### Plasmid Construction for *kojA* Mutation in NSAR1

A *kojA* mutant strain of NSAR1 was created using CRISPR-Cas9 technology, through the construction and application of plasmid pAYargCas9-kojA2.

**Figure 7:**
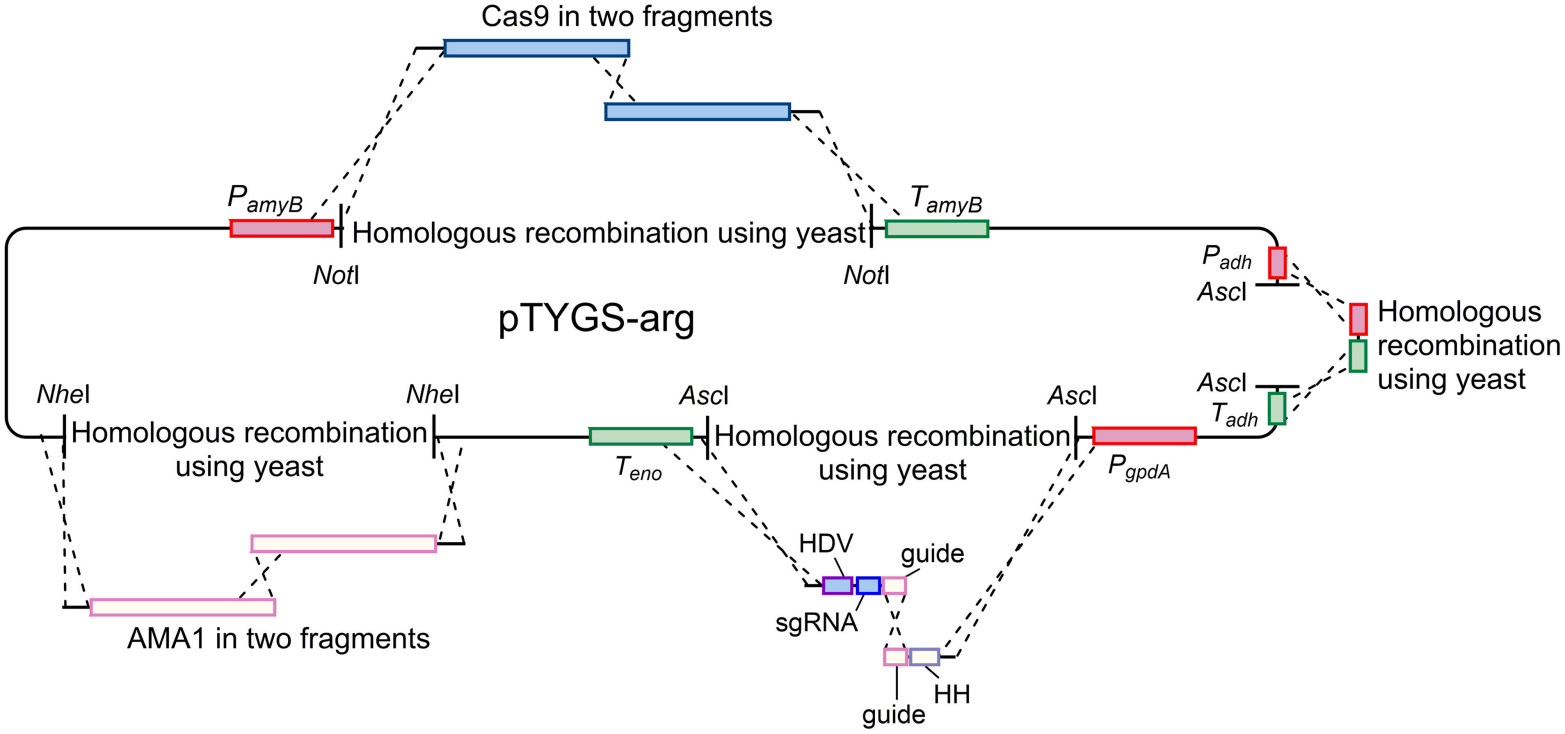
Diagram demonstrating the strategy used to construct the pAYargCas9-kojA2 plasmid. Cas9, AMA1, and the guide RNA were each introduced via consecutive yeast recombinations, as described in the text.

Plasmid pAYargCas9-kojA2 was built from the *A. oryzae* expression plasmid pTYGS-arg (Lazarus et al., 2014) in multiple steps (Figure 7). Firstly, cas9 was recombined into pTYGS-arg by yeast recombination. Cas9 was amplified from plasmid pKWGF2::hCas9 (Nekrasov et al., 2013) in two overlapping fragments, to allow the removal of an internal *Asc*I site, using the primer pairs PamyB-Cas9-F/Cas9-Asc-R and Cas9-Asc-F/Cas9-TamyB-R. These fragments were combined with pTYGS-arg digested with *Not*I, placing *cas9* within the *amyB* cassette and creating plasmid pTYargCas9. pTYargCas9 was then adapted to contain the *AMA1* autonomously replicating sequence to allow for extrachromosomal replication rather than integration, and the subsequent loss of the plasmid to recycle the selectable marker. This was achieved by digesting pTYargCas9 with *NheI* and inserting *AMA1* into this break in two overlapping fragments which were amplified from pFC330 (Nødvig et al., 2015) using the primer pairs AMA1-1F/AMA1-2R and AMA1-2F/AMA1-1R. Again, this was done via yeast recombination, yielding the plasmid pAYargCas9. Finally, the single chimeric guide RNA (sgRNA) was constructed by recombining two PCR products into pAYargCas9 to place them between the *gpdA* promoter and the enolase terminator. This utilized the design of Nødvig and co-workers (Nødvig et al., 2015) where the sgRNA is embedded within a larger transcript from which it is released by the action of two ribozyme sequences; the 5′-end hammerhead (HH) and 3′-end hepatitis delta virus (HDV), which flank the sgRNA. This allows for the use of standard RNA polymerase II promoters, removing the requirement for an RNA polymerase III promoter. Portions of the sgRNA cassette were amplified from plasmid pFC334 (Nødvig et al., 2015) using the primer pairs Pgpd-kojA2-HH-F/Guide-kojA2-R and HH-kojA2-Guide-F/Teno-HDV-R. These primers introduced the necessary *kojA*-specific sequences including the protospacer, the sgRNA backbone and the variable HH sequence. The two resulting PCR products were combined with pAYargCas9 that had been digested with *Asc*I as well as a short PCR product (amplified using primer pair ExAdhSeq.F/ExSeqAdh.R) to repair an *Asc*I break site in another cassette and yeast recombination was conducted (Figure 7). This produced the final plasmid; pAYargCas9-kojA2, which was used to transform *A. oryzae* NSAR1. Primers used in construction of this plasmid are shown in Table 1.

### Screening for Kojic Acid Production From Putative Δ*kojA* Strains

Putative Δ*kojA* transformants were screened by culturing on KA-Fe solid medium (5% (w/v) glucose, 0.25% (w/v) yeast extract, 0.1% (w/v) K_2_HPO_4_, 0.05% (w/v) MgSO4.7H_2_O, 1 mM FeCl_3_, 2% (w/v) agar, pH 5) (Terabayashi et al., 2010). Kojic acid reacts with the ferrous chloride in the medium and causes a clear color change to red. An absence of color change therefore indicates a loss of kojic acid production.

### Genetic Analysis of Transformants

gDNA from nine putative Δ*mcrA A. oryzae* strains was analyzed using 4 sets of primers (Table 1). The first pair tested for integration of the “left hand” flanking region (primer pair AO-mcrA-test-F/pArgB-Bg-R). These primers should only amplify a product if integration has occurred correctly. The second pair (TargB-Nd-F/AO-mcrA-test-R) tested for correct integration of the “right-hand” flanking region. The third pair (AO-mcrA-F/AO-mcrA-R) tested for the presence of the wild-type *Ao_mcrA* gene. If the transformants are genetically pure, then no product should be amplified. As final confirmation, primers AO-mcrA-test-F/AO-mcrA-test-R were used to amplify over the whole disrupted region. Primers used are shown in Table 1. Analytical PCR was performed using Biomix Red (Bioline).

gDNA from *A. oryzae* NSAR1 and the putative Δ*kojA* transformant was analyzed using the primer pair kojA-F/kojA-R (Table 1) which amplified a ~1 kb product. This was subsequently sequenced to identify the 8 bp deletion in the Δ*kojA* transformant. Amplification of fragments for sequencing was performed using the high-fidelity KAPA HiFi HotStart (Roche).

### Analytical HPLC

Sample solutions (20 μl) were analyzed using a Waters LCMS system comprising of a Phenomenex KINETEX column (2.6 μ, C18, 100 Å, 4.6 × 100 mm) equipped with a Phenomenex Security Guard precolumn (Luna C5 300 Å) eluted at 1 ml/min. Detection was by Waters 2998 Diode Array detector between 200 and 600 nm; Waters 2424 ELSD and Waters SQD-2 mass detector operating simultaneously in ES^+^ and ES^−^ modes between 100 and 800 m/z. Solvent A was HPLC grade water containing 0.05% formic acid, and solvent B was HPLC grade acetonitrile containing 0.05% formic acid. Gradient program was as follows: 5–95% of B over 15 min. 0 min, 5% B; 1 min, 5% B; 2 min, 40% B; 15 min, 95% B; 17 min, 95% B; 18 min, 5% B; 20 min, 5% B; flow rate 1 ml/min.

### NMR Analysis

Instruments used; Varian 400-MR (400 MHz), Varian VNMRS500 (500 MHz), Bruker 500 Cryo (500 MHz), or Varian VNMRS600 Cryo (600 MHz). Chemical shifts (δ) quoted in parts per million (ppm) and coupling constants (J) in Hertz (Hz), rounded to 0.5 Hz intervals. Two-dimensional NMR techniques (HSQC, COZY, HMBC) were used routinely for the assignment of structures.

### Metabolic Analysis of Strain *Ao*Δ*mcrA-*7

#### Fermentation and Purification

Initial screens of strain AoΔ*mcrA*-7 were conducted in CMP (3.5% (w/v) Czapek Dox broth, 2% (w/v) maltose, 1% (w/v) peptone), MEB (1.7% (w/v) malt extract, 0.3% (w/v) mycological peptone), PDB (2.4% (w/v) potato dextrose broth), GN [2% (w/v) D(+)-glucose monohydrate, 1% (w/v) Nutrient broth No. 2 (Oxoid, UK)] and rice (50 g of rice, autoclaved in 50 ml of distilled H_2_O). Cultures were grown for 1 week at 28°C with shaking at 180 rpm, with the exception of the rice culture which is a static culture (28°C). Cultures were extracted with an equal volume of ethyl acetate and analyzed by LCMS.

For purification of compounds, strain AoΔ*mcrA*-7 was cultured for 3 days in CMP medium at 28°C. This seed culture was then used to inoculate 2 l of CMP. The flasks were shaken at 180 rpm at 28°C for 1 week. The broth was filtered by Buchner filtration and extracted with ethyl acetate (1.5 l × 3). The organic layers were concentrated under reduced pressure to give 7.4 g of crude extract. The major product **1** (3.6 g) was crystallized out from ethyl acetate. The mother liquor was then chromatographed by reversed-phase silica gel column chromatography eluted with acetonitrile/water (v/v, from 1:3 to 1:0) to yield 3 fractions (F1–F3). From fraction F2 (320 mg), **2** (7.4 mg), a mixture of **3** and **4** (13.5 mg), **5** (3.8 mg), **6** (2.5 mg), and **7** (3.5 mg) were afforded by preparative LCMS (Luna 5μ C_18_ 100 Å, 250 × 10 mm, 8 ml/min) using a 20 min 5–60% acetonitrile in water (+ 0.1% formic acid).

#### Isolated Metabolites

Kojic acid (**1**), white crystals, UV λ_max_ 216, 268 nm, ESI-MS *m/z* 143 [M + H]^+^. ^1^H NMR (500 MHz, CD_3_OD, δ_H_): 4.41 (2H, s, H-6), 6.50 (1H, s, H-4), 7.96 (1H, s, H-1); δ_C_ (125 MHz, CD_3_OD) 61.4 (C-6), 110.9 (C-4), 141.2 (C-1), 147.6 (C-2), 170.6 (C-5), 177.1 (C-3). These data were in agreement with the reported literature (Kingsbury et al., 1976).

Kojic acid dimer (**2**), white amorphous powder, UV λ_max_ 216, 273 nm, ESI-MS *m/z* 283 [M + H]^+^. ^1^H NMR (500 MHz, CD_3_OD, δ_H_): 4.35 (1H, d, *J* = 12.0 Hz, H-12a), 4.41 (1H, d, *J* = 12.0 Hz, H-12b), 5.94 (1H, s, H-6), 6.49 (1H, s, H-10), 6.73 (1H, s, H-4), 7.94 (1H, s, H-1); δ_C_ (125 MHz, CD_3_OD) 61.1 (C-12), 65.9 (C-6), 110.1 (C-10), 111.7 (C-4), 141.2 (C-1), 144.5 (C-8), 147.6 (C-2), 147.9 (C-7), 168.1 (C-5), 170.1 (C-11), 176.6 (C-9), 176.7 (C-3). These data were in agreement with the reported literature (Nakagawa et al., 1993).

A mixture (1:1) of compounds **3** and **4** was obtained as a white amorphous powder. *Cyclo*-[(_D_)Pro-(L)Tyr] (**3**), ESI-MS *m/z* 261 [M + H]^+^. ^1^H NMR (500 MHz, CD_3_OD, δ_H_): 1.95 (2H, m, H-3a and 4a), 2.04 (1H, m, H-4b), 2.33 (1H, m, H-3b), 3.07 (2H, m, H-8), 3.57 (2H, m, H-5), 4.22 (1H, m, H-2), 4.38 (1H, m, H-7), 6.72 (2H, d, *J* = 8.5 Hz, H-11 and H-13), 7.04 (2H, d, *J* = 8.5 Hz, H-10 and H-14); δ_C_ (125 MHz, CD_3_OD) 23.3 (C-4), 29.5 (C-3), 37.7 (C-8), 46.2 (C-5), 57.9 (C-7), 60.1 (C-2), 116.2 (C-11 and C-13), 127.6 (C-9), 132.1 (C-10), 157.7 (C-12), 166.9 (C-6), 172.5 (C-1). These data were in agreement with the reported literature (Kumar et al., 2012).

*Cyclo*-[(_D_)Pro-(_L_)Val] (**4**), ESI-MS *m/z* 197 [M + H]^+^. ^1^H NMR (500 MHz, CD_3_OD, δ_H_): 0.95 (3H, d, *J* = 7.5 Hz, H-9), 1.11 (3H, d, *J* = 7.5 Hz, H-10), 1.24 (1H, m, H-3a), 1.81 (2H, m, H-4), 2.11 (1H, m, H-3b), 2.51 (1H, m, H-8), 3.36 (1H, m, H-5a), 3.52 (1H, m, H-5b), 4.05 (1H, m, H-7), 4.07 (1H, m, H-2); δ_C_ (125 MHz, CD_3_OD) 16.7 (C-9), 18.4 (C-10), 22.7 (C-4), 29.4 (C-3), 29.9 (C-8), 45.9 (C-5), 60.1 (C-2), 61.5 (C-7), 167.6 (C-6), 170.7 (C-1). These data were in agreement with the reported literature (Kwon et al., 2001).

*Cyclo*-[(_D_)Pro-(_L_)Ile] (**5**), white amorphous powder, [α] + 72° (*c* 0.01, MeOH), ESI-MS *m/z* 211 [M + H]^+^. ^1^H NMR (500 MHz, CD_3_OD, δ_H_): 0.96 (3H, d, *J* = 7.5 Hz, H-10), 1.08 (3H, d, *J* = 7.5 Hz, H-11), 1.34 (1H, m, H-9a), 1.47 (1H, m, H-9b), 1.95 (2H, m, H-3a and H-4a), 2.04 (1H, m, H-4b), 2.34 (1H, m, H-3b), 3.07 (1H, m, H-8), 3.50-3.58 (2H, m, H-5), 4.09 (1H, m, H-7), 4.21 (1H, m, H-2); δ_C_ (125 MHz, CD_3_OD) 12.6 (C-10), 15.5 (C-11), 23.2 (C-4), 25.4 (C-9), 29.6 (C-3), 37.1 (C-8), 60.0 (C-2), 61.3 (C-7), 167.5 (C-6), 172.4 (C-1). These data were in agreement with the reported literature (Bull et al., 1998).

*Cyclo*-[(_D_)Pro-(_L_)Leu] (**6**), white amorphous powder, [α] – 142° (*c* 0.01, MeOH), UV λ_max_ 210 nm, ESI-MS *m/z* 211 [M + H]^+^. ^1^H NMR (500 MHz, CD_3_OD, δ_H_): 0.97 (6H, m, H-10 and H-11), 1.53 (1H, m, H-8a), 1.91 (2H, m, H-4a and H-9), 1.95 (1H, m, H-9b), 2.03 (1H, m, H-4b), 2.31 (1H, m, H-3b), 3.53 (1H, m, H-5), 4.14 (1H, m, H-7), 4.27 (1H, m, H-2); δ_C_ (125 MHz, CD_3_OD) 20.8 (C-10), 21.9 (C-11), 22.2 (C-4), 24.4 (C-9), 27.7 (C-3), 37.9 (C-8), 45.0 (C-5), 53.2 (C-7), 58.9 (C-2), 167.5 (C-6), 171.3 (C-1). These data were in agreement with the reported literature (Kumar et al., 2012).

*Cyclo*-[(_D_)Pro-(_L_)Phe] (**7**), white amorphous powder, [α] + 51° (*c* 0.01, MeOH), UV λ_max_ 210 nm, ESI-MS *m/z* 245 [M + H]^+^. ^1^H NMR (500 MHz, CD_3_OD, δ_H_): 1.24 (1H, m, H-3a), 1.81 (2H, m, H-4), 2.11 (1H, m, H-3a), 3.19 (2H, m, H-8), 3.40 (1H, m, H-5a), 3.54 (1H, m, H-5b), 4.10 (1H, m, H-2), 4.45 (1H, m, H-7), 7.23 – 7.31 (5H, m, H-10, 11, 12, 13, 14); δ_C_ (125 MHz, CD_3_OD) 22.8 (C-4), 29.4 (C-3), 38.2 (C-8), 45.9 (C-5), 57.7 (C-7), 60.1 (C-2), 128.1 (C-12), 129.4 (C-11 and C-13), 131.0 (C-10 and C-14), 137.3 (C-9), 166.8 (C-6), 170.9 (C-1). These data were in agreement with the reported literature (Kumar et al., 2013).

### Fermentation and Chemical Analysis of Tenellin Producing Strains

Strains ΔK-Ten120 and N-Ten402 were cultured on solid CMP medium (3.5% (w/v) Czapek Dox broth, 2% (w/v) maltose, 1% (w/v) peptone, 2% (w/v) agar) for 5 days at 28°C. Subsequently, 1 cm^2^ plugs of agar from the rim and the center of growing colonies were extracted in 2 ml of a solution of ethyl acetate, dichloromethane and methanol (3:2:1) with 1% acetic acid. After 1 h in an ultrasonic bath, the liquid was removed and dried. The crude extract was then dissolved in 200 μl acetonitrile and analyzed by HPLC-MS/UV/ELSD.

### Kojic Acid Quantitative Analysis

#### Kojic Acid Calibration Curve

Acetonitrile solutions containing specific kojic acid concentrations were prepared (0.25–1.5 mg/ml) and injected into our HPLC system in triplicate. The calibration curve was created by regression analysis plotting the kojic acid concentrations (mg/ml) with the area of the peak obtained from the ELSD chromatogram. The relationship was linear within the 0.25–1.50 mg/ml concentration range and the lower limit of detection was 0.20 mg/ml (Supplementary Figure 46). The obtained linear regression equation, *y* = 18784*x* – 5527.7 (with the coefficient of correlation *r*^2^ = 0.98), was then applied to quantify kojic acid in the crude extracts. All analyses were performed in triplicate, and the statistical analysis of the data was achieved by using Microsoft Office Excel 2016.

#### Sample Preparation

Strains *A. oryzae* NSAR1 and AoΔ*mcrA*-7 were cultured for 2 days in CMP media at 28°C. From this seeding culture, 200 μl was used to inoculate conical flasks containing 50 ml of CMP. The flasks were then shaken at 180 rpm at 28°C for 1 week. The broths were separated from the mycelium, and a 10 ml portion of each culture was extracted with ethyl acetate (10 ml) by shaking for 3 min. A 5 ml portion of the organic layer was then concentrated under nitrogen gas to give a crude extract. The crude extract was then dissolved in acetonitrile at the concentration of 1 mg/ml for HPLC injection. Each extraction was performed in triplicate.

## Data Availability Statement

The original contributions generated for the study are included in the article/Supplementary Material, further inquiries can be directed to the corresponding author/s.

## Author Contributions

AB and CL contributed to the design of the research. CL contributed to plasmid design and construction. TD, KdM-S, IP, KW, and MZ performed the experimental work. TD, KdM-S, IP, KW, and CW were involved in interpretation of data. KdM-S and KW drafted the manuscript. CL, CW, and AB had supervisory roles. All authors contributed to the article and approved the submitted version.

## Conflict of Interest

The authors declare that the research was conducted in the absence of any commercial or financial relationships that could be construed as a potential conflict of interest.

## Abbreviations

SM: Secondary Metabolite
GRAS: Generally Recognized As Safe
*mcrA*: multicluster regulator A
BGC: Biosynthetic Gene Cluster
PCR: Polymerase Chain Reaction
LCMS: Liquid Chromatography Mass Spectrometry
ELSD: Evaporative Light Scattering Detector
HPLC: High Pressure Liquid Chromatography
TLC: Thin-Layer Chromatography
CRISPR, Clustered: Regularly Interspaced Short Palindromic Repeats
Cas: CRISPR-associated proteins
FAD: Flavin adenine dinucleotide
UV: Ultraviolet
ESI-MS: Electrospray ionization - Mass Spectrometry
NMR: Nuclear magnetic resonance
*P*_*amyB*_: promoter for *A. oryzae* α-amylase
*P*_*adh*_: promoter for *A. oryzae* alcohol dehydrogenase
*P*_*gpdA*_: promoter for *Aspergillus nidulans* glyceraldehyde-3-phosphate dehydrogenase
*P*_*eno*_: promoter for *A. oryzae* enolase
*argB*: *Aspergillus nidulans* ornithine carbamoyltransferase gene
*AMA1*: autonomously maintained in *Aspergillus*
RNA: Ribonucleic acid
sgRNA: single chimeric guide RNA
HH: 5′-end hammerhead ribozyme sequence
HDV: 3′-end hepatitis delta virus ribozyme sequence
rpm: revolutions per minute.
