# Supplementary material for: Cleaning the Cellular Factory–Deletion of McrA in Aspergillus oryzae NSAR1 and the Generation of a Novel Kojic Acid Deficient Strain for Cleaner Heterologous Production of Secondary Metabolites
Source: Front Fungal Biol. 2021 Feb 9;2:632542. doi: 10.3389/ffunb.2021.632542 (PMC10512265; doi:10.3389/ffunb.2021.632542)
Supplement: Supplementary file 1 [file Data_Sheet_1.PDF]

frontiers

Supplementary Material

Cleaning the Cellular Factory – Deletion of McrA in *Aspergillus oryzae* NSAR1 and the generation of a novel kojic acid deficient strain for cleaner heterologous production of secondary metabolites

Trong T. Dao[2†], Kate M. J. de Mattos-Shipley[1†], Ian M. Prosser[1†], Katherine Williams[1†], Marija K. Zacharova[2], Colin M. Lazarus[1], Christine L. Willis[2] and Andrew M. Bailey[1*]

1: School of Biological Sciences, Bristol Life Sciences Building, University of Bristol, 24 Tyndall Ave, Bristol, BS8 1TQ, UK

2: School of Chemistry, University of Bristol, Cantock's Close, Bristol, BS8 1TS, UK

[†] All four authors should be considered joint first authors

* Correspondence: Andy.Bailey@bristol.ac.uk

Table of Figures

Supplementary Figure 1: An alignment of the three putative *A. oryzae* McrA protein sequences....................... 3
Supplementary Figure 2: An alignment of the characterised McrA protein from *A. nidulans*............................ 4
Supplementary Figure 3: Analytical PCRs conducted for putative *Ao_mcrA* knock-out strains 5
Supplementary Figure 4: HPLC traces (ELSD) for purified compounds **1 - 7** 6
Supplementary Figure 5: UV spectrum of **1**. 7
Supplementary Figure 6: Mass spectrum (ES^+) of **1**... 8
Supplementary Figure 7: ^{1}H NMR spectrum (CD_3OD, 500 MHz) of **1**. 8
Supplementary Figure 8: ^{13}C NMR spectrum (CD_3OD, 125 MHz) of **1**. 9
Supplementary Figure 9: UV spectrum of **2** 9
Supplementary Figure 10: Mass spectrum (ES^+) of **2**... 10
Supplementary Figure 11: ^{1}H NMR spectrum (CD_3OD, 500 MHz) of **2** 10
Supplementary Figure 12: ^{13}C NMR spectrum (CD_3OD, 125 MHz) of **2**. 11
Supplementary Figure 13: HSQC spectrum of **2** 11
Supplementary Figure 14: HMBC spectrum of **2** 12
Supplementary Figure 15: UV spectrum of the mixture **3** and **4** 12
Supplementary Figure 16: Mass spectrum (ES^+) of the mixture **3** and **4** 13
Supplementary Figure 17: ^{1}H NMR spectrum (CD_3OD, 500 MHz) of the mixture **3** and **4**............................. 13
Supplementary Figure 18: ^{13}C NMR spectrum (CD_3OD, 125 MHz) of the mixture **3** and **4**. 14
Supplementary Figure 19: COSY spectrum of the mixture **3** and **4** 14
Supplementary Figure 20: HSQC spectrum of the mixture **3** and **4** 15
Supplementary Figure 21: HMBC spectrum of the mixture **3** and **4** 15
Supplementary Figure 22: UV spectrum of **5** 16
Supplementary Figure 23: Mass spectrum (ES^+) of **5**... 16
Supplementary Figure 24: ^{1}H NMR spectrum (CD_3OD, 125 MHz) of **5**. 17
Supplementary Figure 25: ^{13}C NMR spectrum (CD_3OD, 125 MHz) of **5**. 17
Supplementary Figure 26: COSY spectrum of **5** 18
Supplementary Figure 27: HSQC spectrum of **5** 18
Supplementary Figure 28: HMBC spectrum of **5** 19
Supplementary Figure 29: UV spectrum of **6** 20
Supplementary Figure 30: Mass spectrum (ES^+) of **6**... 20
Supplementary Figure 31: ^{1}H NMR spectrum (CD_3OD, 125 MHz) of **6** 21
Supplementary Figure 32: ^{13}C NMR spectrum (CD_3OD, 125 MHz) of **6**. 21
Supplementary Figure 33: COSY spectrum of **6** 22
Supplementary Figure 34: HSQC spectrum of **6** 22
Supplementary Figure 35: HMBC spectrum of **6** 23
Supplementary Figure 36: UV spectrum of **7** 24
Supplementary Figure 37: Mass spectrum (ES^+) of **7**... 24
Supplementary Figure 38: ^{1}H NMR spectrum (CD_3OD, 500 MHz) of **7**. 25
Supplementary Figure 39: ^{13}C NMR spectrum (CD_3OD, 125 MHz) of **7**. 25
Supplementary Figure 40: COSY spectrum of **7** 26
Supplementary Figure 41: HSQC spectrum of **7** 26
Supplementary Figure 42: HMBC spectrum of **7**. 27
Supplementary Figure 43: LCMS comparison of AoΔ*mcrA*-7 and CMP media 28
Supplementary Figure 44: TLC analysis of NSARΔK.. 28
Supplementary Figure 45: HPLC analysis of NSARΔK 29
Supplementary Figure 46: Calibration curve for the quantification of kojic acid 30

Supplementary Figures

```
EIT82532.1        MSSNPHDFSDPNRQGQYPPPQWNTSQPEDNPSAHYPPASQYPYPPASYPPPSADHQYPPP      60
OOO05597.1        ------------------------------------------------------------      0
XP_023092425.1    ------------------------------------------------------------      0

EIT82532.1        PQSQYPPPPNMAAIHPHVQGPQDPYRLPPPPGAYRPPDVYAQPPPPQVVYQAAAPRQRTA      120
OOO05597.1        ----------MAAIHPHVQGPQDPYRLPPPPGAYRPPDVYAQPPPPQVVYQAAAPRQRTA      50
XP_023092425.1    ------------------------------------------------------------      0

EIT82532.1        IACRYCRRRKIRCSGFESSQDGRCSNCIRFNQECMFTPVSSQAQAFVPAHAAYPHLRNAQ      180
OOO05597.1        IACRYCRRRKIRCSGFESSQDGRCSNCIRFNQECMFTPVSSQAQAFVPAHAAYPHLRNAQ      110
XP_023092425.1    ----------------------------------MFTPVSSQAQAFVPAHAAYPHLRNAQ      26
                                                  **************************

EIT82532.1        NQPRGGAPVMLYGAHGQPLPPQQQPQAPPDATLPPPQGLYQNPYGSAPPPLPQDP-----      235
OOO05597.1        NQPRGGAPVMLYGAHGQPLPPQQQPQAPPDATLPPPQGLYQNPYGSAPPPLPQDPVSSNS      170
XP_023092425.1    NQPRGGAPVMLYGAHGQPLPPQQQPQAPPDATLPPPQGLYQNPYGSAPPPLPQDPVSSNS      86
                  *******************************************************

EIT82532.1        -----------------RPIGRRGSSSGFEYPDPTNLAPVTPATSAPGYQAHSASSPYYP      278
OOO05597.1        ALSAVKSTLTHWSLCEQRPIGRRGSSSGFEYPDPTNLAPVTPATSAPGYQAHSASSPYYP      230
XP_023092425.1    ALSAVKSTLTHWSLCEQRPIGRRGSSSGFEYPDPTNLAPVTPATSAPGYQAHSASSPYYP      146
                                   *******************************************

EIT82532.1        PPPQHDRRPSPQSAYPYDNRHSSSPHNSPYPPLHASQSAMTPPPTSTPGGSSRGGLNVRD      338
OOO05597.1        PPPQHDRRPSPQSAYPYDNRHSSSPHNSPYPPLHASQSAMTPPPTSTPGGSSRGGLNVRD      290
XP_023092425.1    PPPQHDRRPSPQSAYPYDNRHSSSPHNSPYPPLHASQSAMTPPPTSTPGGSSRGGLNVRD      206
                  ************************************************************

EIT82532.1        MLNPGDSQGRSSTDSDMLNALNRRGLNQ         366
OOO05597.1        MLNPGDSQGRSSTDSDMLNALNRRGLNQ         318
XP_023092425.1    MLNPGDSQGRSSTDSDMLNALNRRGLNQ         234
                  ****************************
```

Supplementary Figure 1: An alignment of the three putative *A. oryzae* McrA protein sequences identified from the NCBI database, from *A. oryzae* strains 3.042 (accession number EIT82532.1), BCC7051 (accession number OOO05597.1) and RIB40 (accession number XP_023092425.1). A GAL4-like Zn2Cys6 binuclear cluster DNA-binding domain (accession number cd00067) is shown in red.

```
A.nidulans-McrA    MSNNPNPSNPNDYNDPSRRGQYPPPQWNSSQPEESPS-PYPPASQYPYPPSSYPPASYPP   59
A.oryzae-McrA      -----MSSNPHDFSDPNRQGQYPPPQWNTSQPEDNPSAHYPPASQYPYPPASY-----PP   50
                          ***:*:.**.*:*********:****:.**  ***********:**     **

A.nidulans-McrA    PAEAHQYPPPPAHYPPPQSQYPPQSQYPPPQSNMASVHP-MHPQQPPYQLPPPPGAYRPD   118
A.oryzae-McrA      PSADHQYPPPP------------QSQYPPPP-NMAAIHPHVQGPQDPYRLPPPPGAYRPP   97
                   *:  *******            *******  ***::** ::  * **:**********

A.nidulans-McrA    VAYGQQPPPPPPPYGQQPPPSGAPAPGGVVYQAAPRQRTAIACRYCRRRKIRCSGFEHSS   178
A.oryzae-McrA      DVYAQPPPP-----------------QVVYQAAAPRQRTAIACRYCRRRKIRCSGFESSQ   140
                    .*.* ***                   *   ************************* *.

A.nidulans-McrA    DGRCTNCVRFSQQCVFTPVSSQAQAFVPVQAAYPHLRNGQNG-RPGEPVVLYGAHGQPLP   237
A.oryzae-McrA      DGRCSNCIRFNQECMFTPVSSQAQAFVPAHAAYPHLRNAQNQPRGGAPVMLYGAHGQPLP   200
                   ****:**:**.*:*:*************.:********.**  * * **:**********

A.nidulans-McrA    PQQQPQAGPETTLPPPQGMYQHHYGGATPPLASVPQDHRPRRGSGSGYDYPDPTNLAPVT   297
A.oryzae-McrA      PQQQPQAPPDATLPPPQGLYQNPYGSAPPPLPQDPRP-IGRRGSSSGFEYPDPTNLAPVT   259
                   ******* *::*******:**: **.* *** . *:    ****.**::***********

A.nidulans-McrA    PVTSAPYQP---RAAPYF-PPPPHDRRPSPQGAYSYDNRHSSSPHASPYPGMHPQN-GAT   352
A.oryzae-McrA      PATSAPGYQAHSASSPYYPPPPQHDRRPSPQSAYPYDNRHSSSPHNSPYPPLHASQSAMT   319
                   *.****       ::**: *** ********.** ********** **** :* .: . *

A.nidulans-McrA    PPPTSTPGSAPRNGLNVRDMLNPGENPGRSSTDSDMLNALDRRGLGQ                399
A.oryzae-McrA      PPPTSTPGGSSRGGLNVRDMLNPGDSQGRSSTDSDMLNALNRRGLNQ                366
                   ********.: *.***********:. *************:****.*
```

Supplementary Figure 2: An alignment of the characterised McrA protein from *A. nidulans* (accession number AN8694) and the *A. oryzae* homologue (accession number EIT82532.1), which is present in the three *A. oryzae* genomes investigated (strains 3.042, BCC7051 and RIB40). A GAL4-like Zn2Cys6 binuclear cluster DNA-binding domain (accession number cd00067) is shown in red.

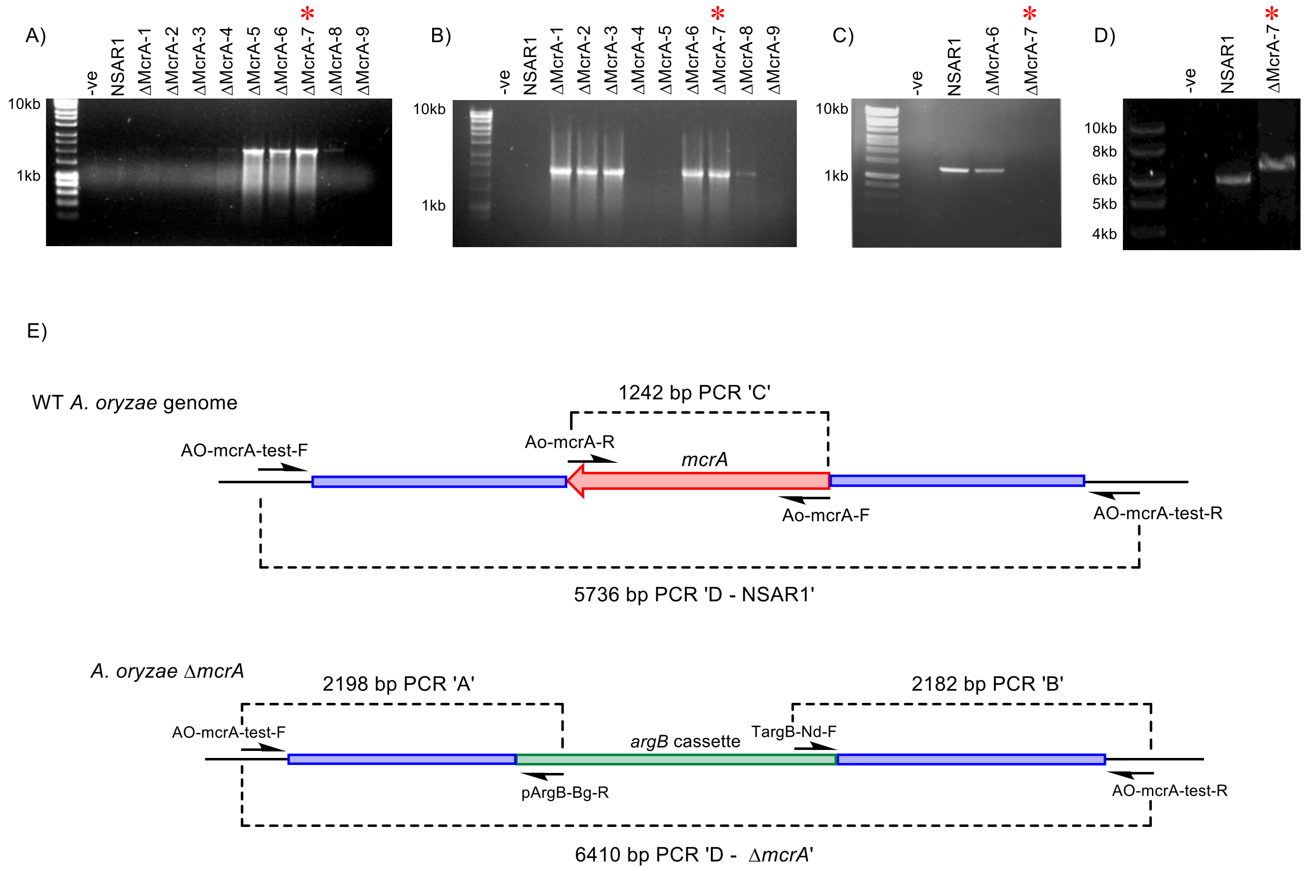

Supplementary Figure 3: Analytical PCRs conducted for putative *Ao_mcrA* knock-out strains. Each gel shows a negative control (lane 2; H_2O), an NSAR1 'WT' control (lane 3), and a range of individual transformants (lanes 4+). **A**) PCR with primers AO-mcrA-test-F and pArgB-Bg-R, testing for correct integration of the knock-out construct at the left boundary of *Ao_mcrA*. **B)** PCR with primers TargB-Nd-F and AO-mcrA-test-R, testing for correct integration of the knock-out construct at the right boundary of *Ao_mcrA*. **C)** PCR with primers AO-mcrA-F and AO-mcrA-R, testing for the presence or absence of the wild-type *Ao_mcrA* gene. **D**) PCR with primers AO-mcrA-test-F and AO-mcrA-test-R, which differentiate between the size of the targeted locus in 'WT' NSAR1 vs. *Ao_mcrA* knock-out strains (~5.7 kb vs. ~6.4 kb respectively). **E**) A schematic showing the primer binding sites for the different diagnostic PCRs and how the product sizes differ based on whether the genotype is 'wildtype (WT)' *A. oryzae* NSAR1 or an *Ao_mcrA* knock-out strain.
N.B. This analysis does not rule out the possibility of additional ectopic integrations of pE-YA-mcrAKO.

These diagnostic PCRs confirm targeted integration of the knock-out construct for strains *AoΔmcrA*-6 and *AoΔmcrA*-7 (Supplementary Figure 3A, 3B), but show that *AoΔmcrA*-6 still retains a wild-type copy of McrA, a scenario which is possible due to the multinucleate nature of *Aspergillus* fungi. Transformant *AoΔmcrA*-7 (highlighted with *) passed all diagnostic screens, with both correct integration and confirmation of genetic purity due to the absence of any wild-type *mcrA* (Supplementary Fig. 3C).

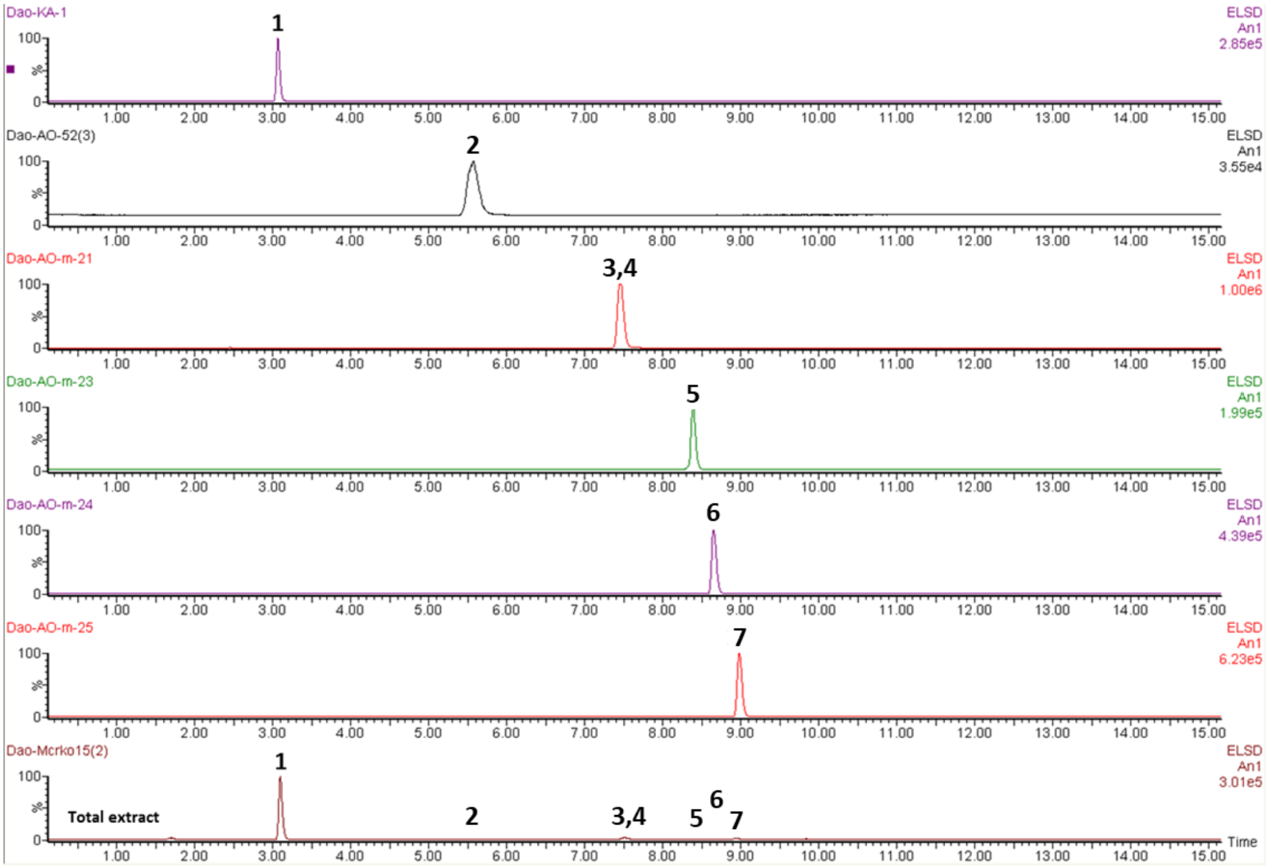

Supplementary Figure 4: HPLC traces (ELSD) for purified compounds **1** - **7** which were isolated from a crude extract of *A. oryzae* strain AoΔ*mcrA*-7 (bottom trace) cultured in CMP.

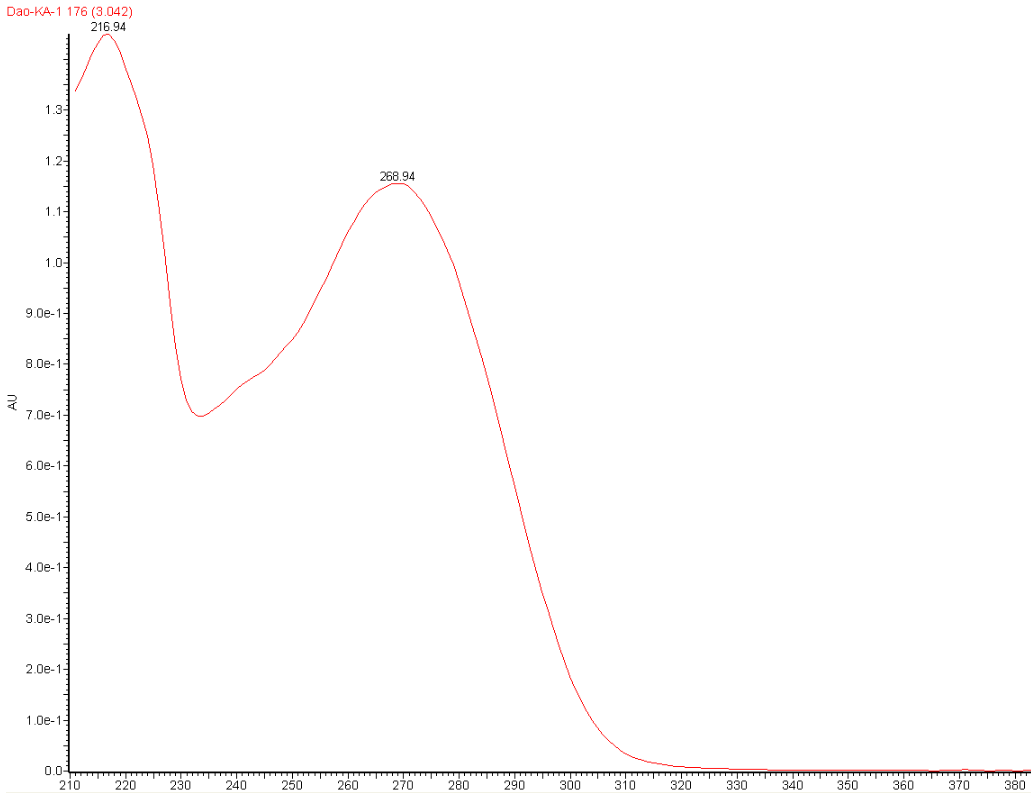

Supplementary Figure 5: UV spectrum of **1**.

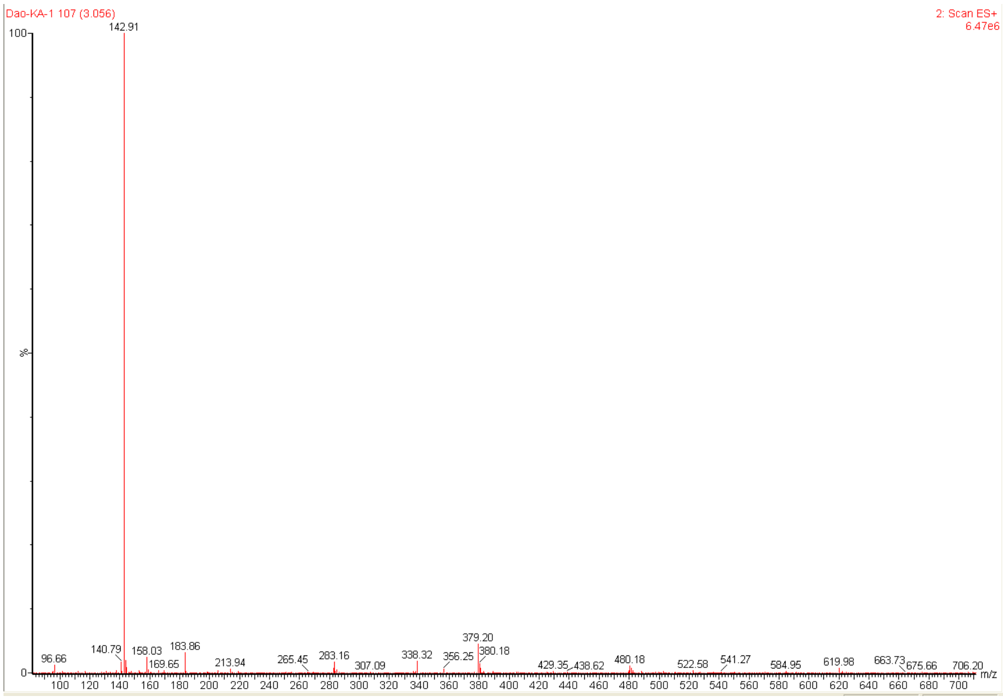

Supplementary Figure 6: Mass spectrum (ES^+) of **1**.

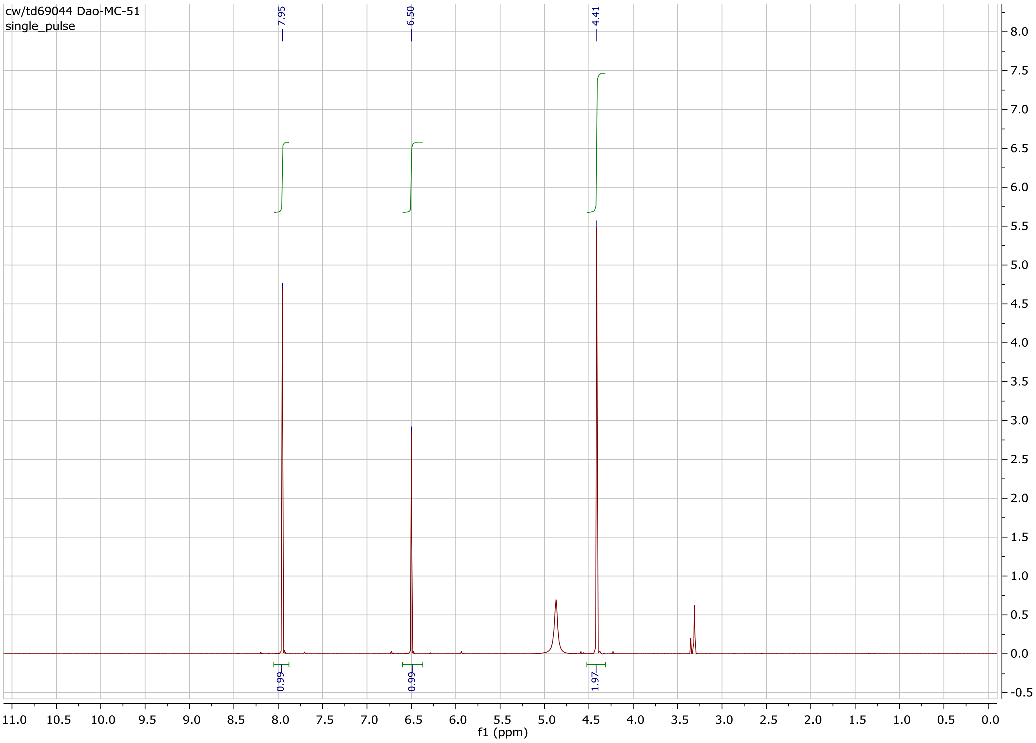

Supplementary Figure 7: ^1H NMR spectrum (CD_3OD, 500 MHz) of **1**.

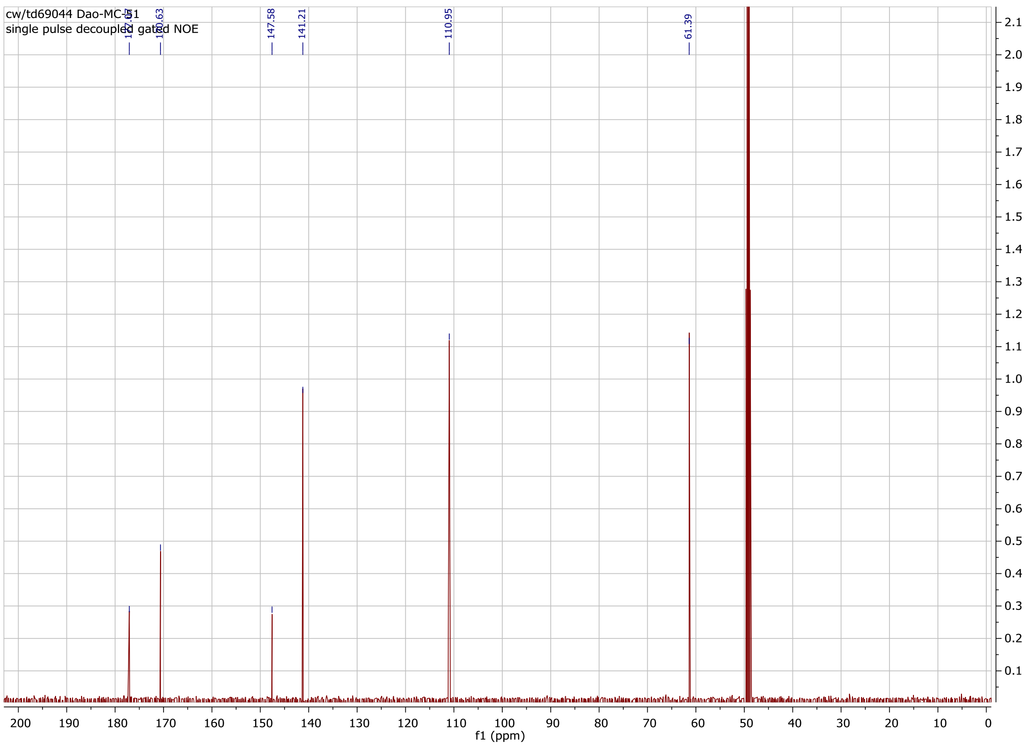

Supplementary Figure 8: ^{13}C NMR spectrum (CD_3OD, 125 MHz) of **1**.

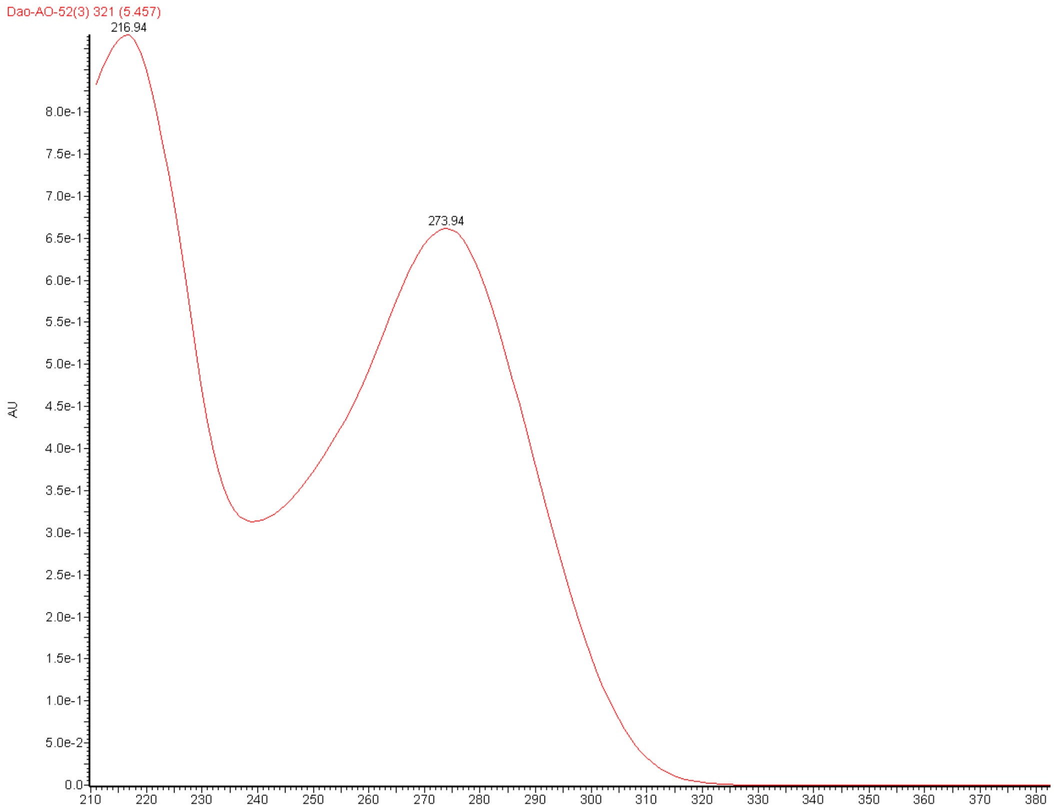

Supplementary Figure 9: UV spectrum of **2**.

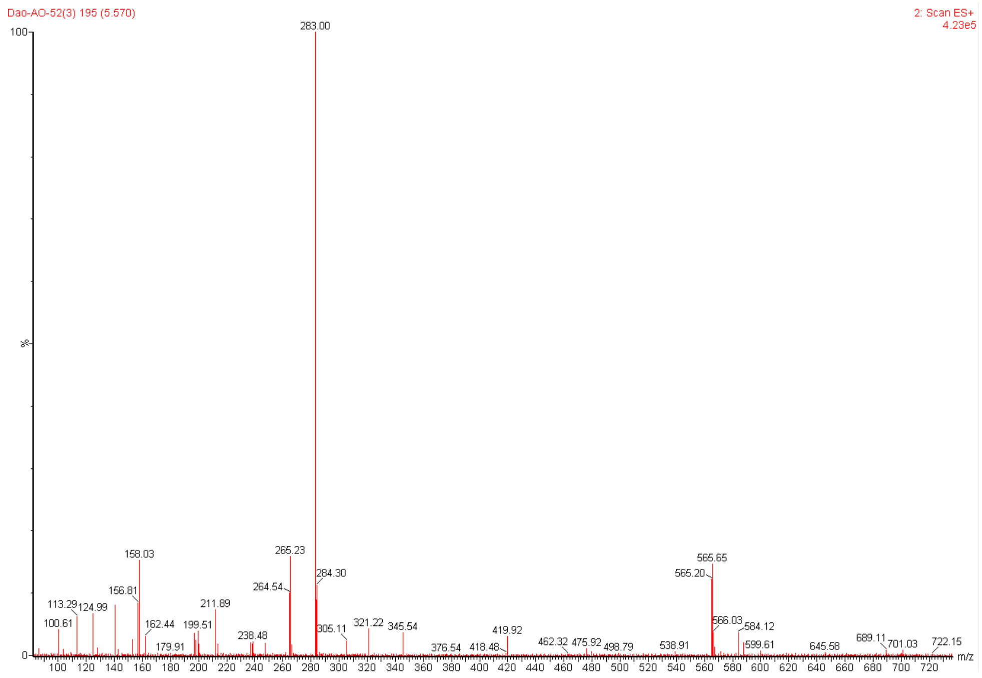

Supplementary Figure 10: Mass spectrum (ES^+) of **2**.

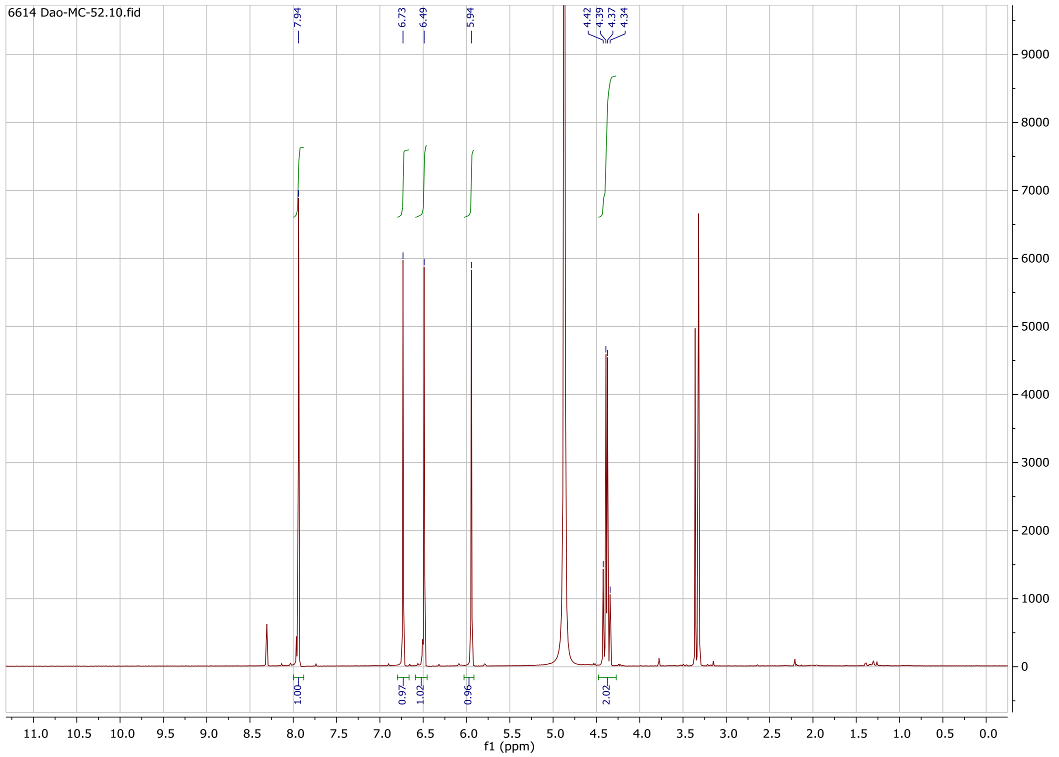

Supplementary Figure 11: ^{1}H NMR spectrum (CD_3OD, 500 MHz) of **2**.

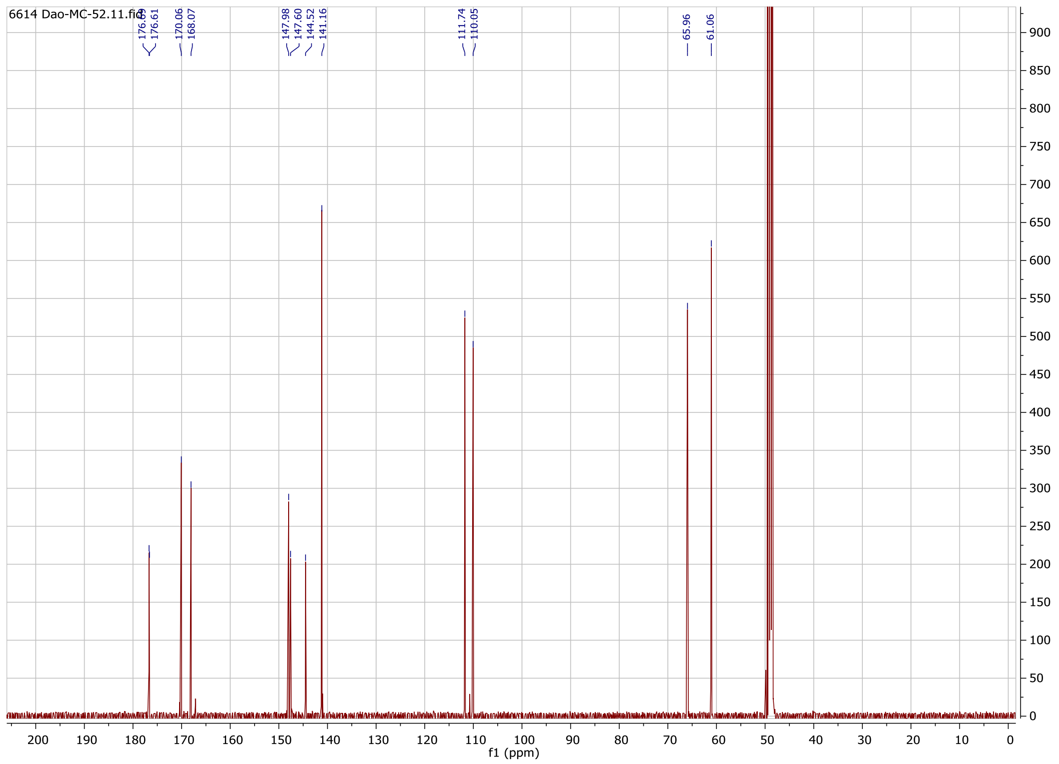

Supplementary Figure 12: ^{13}C NMR spectrum (CD_3OD, 125 MHz) of **2**.

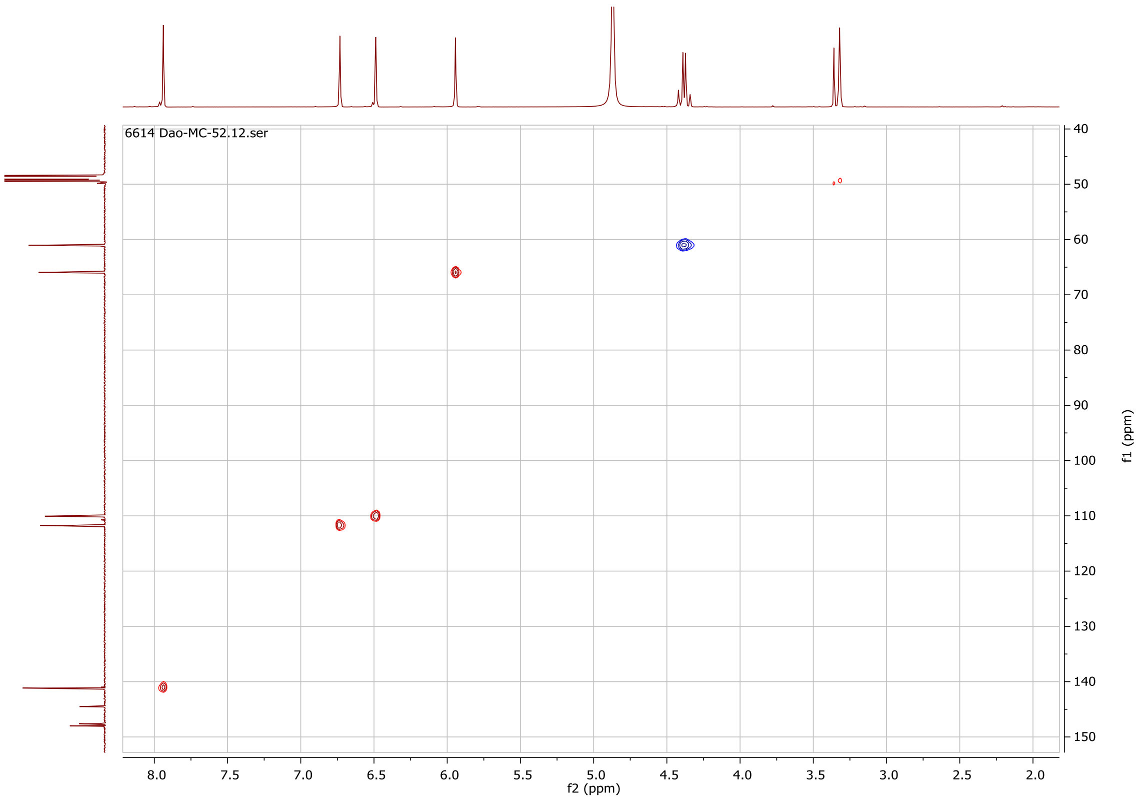

Supplementary Figure 13: HSQC spectrum of **2**.

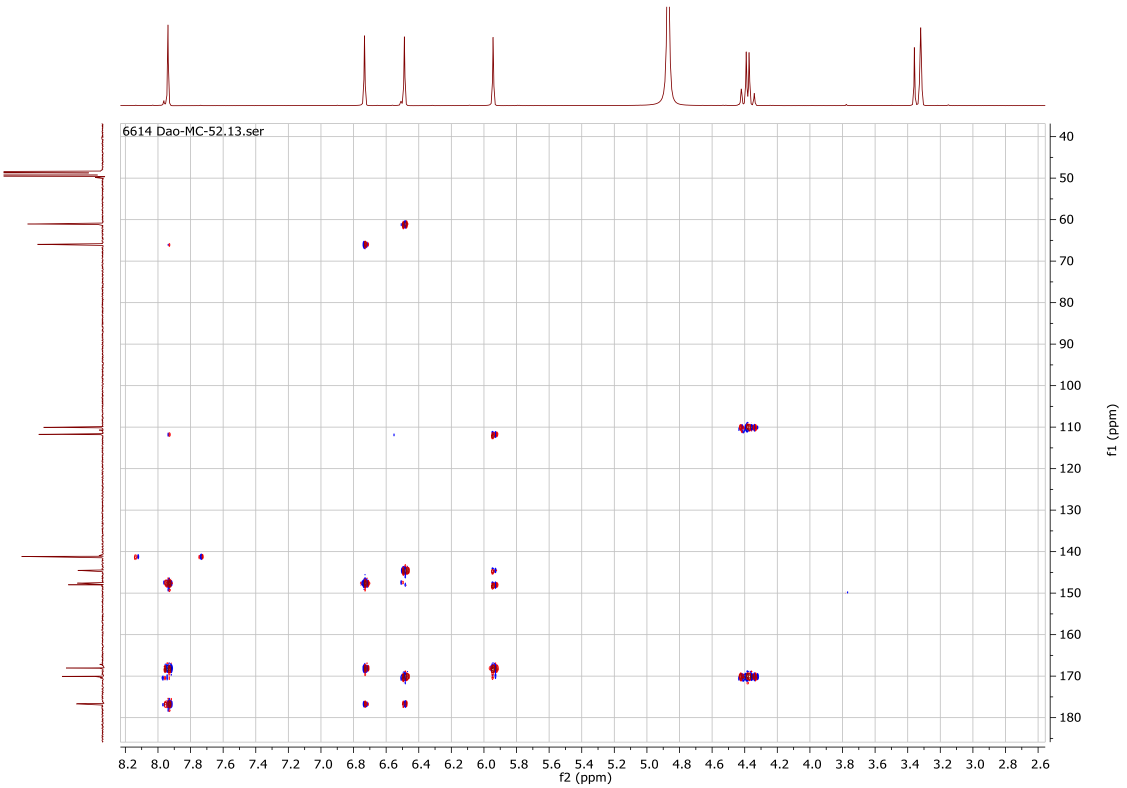

Supplementary Figure 14: HMBC spectrum of **2**.

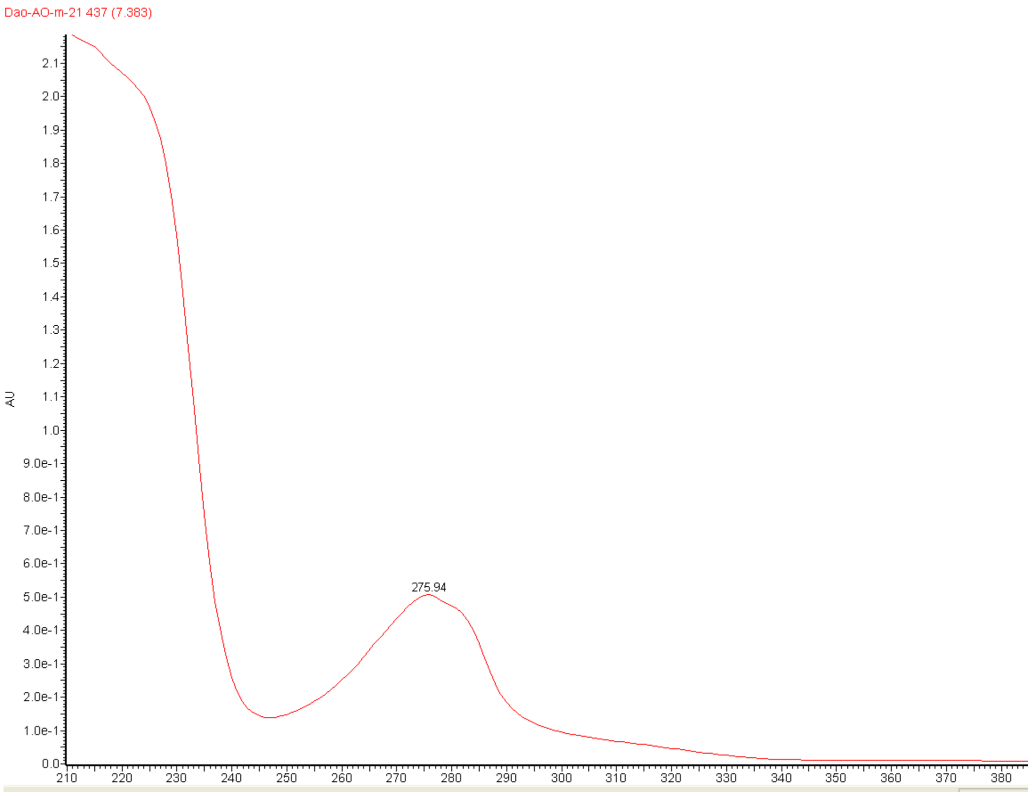

Supplementary Figure 15: UV spectrum of the mixture **3** and **4**.

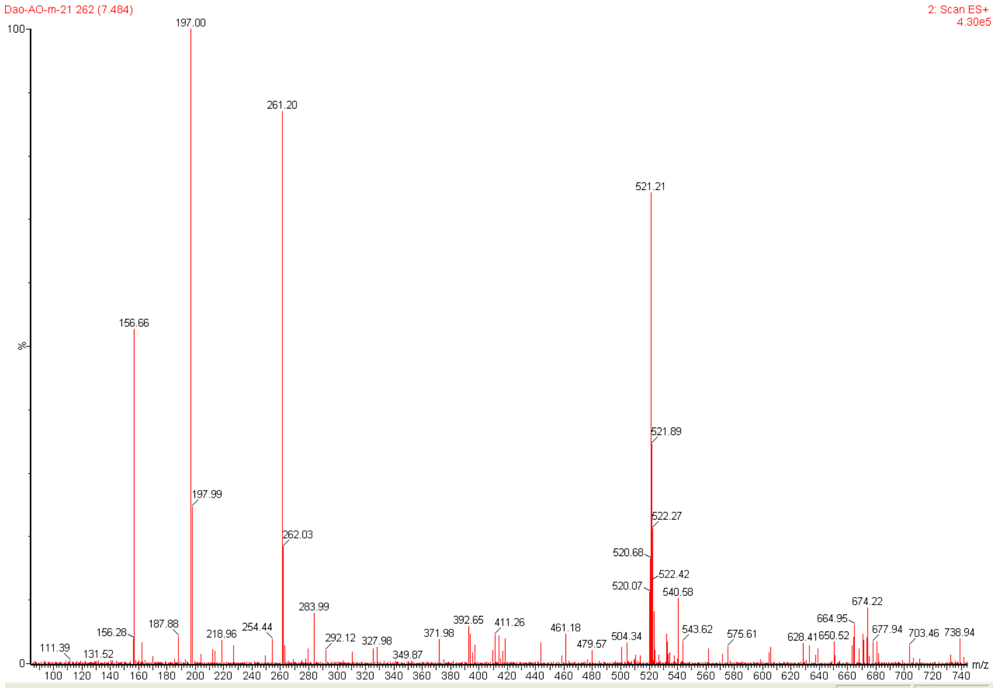

Supplementary Figure 16: Mass spectrum (ES^+) of the mixture **3** and **4**.

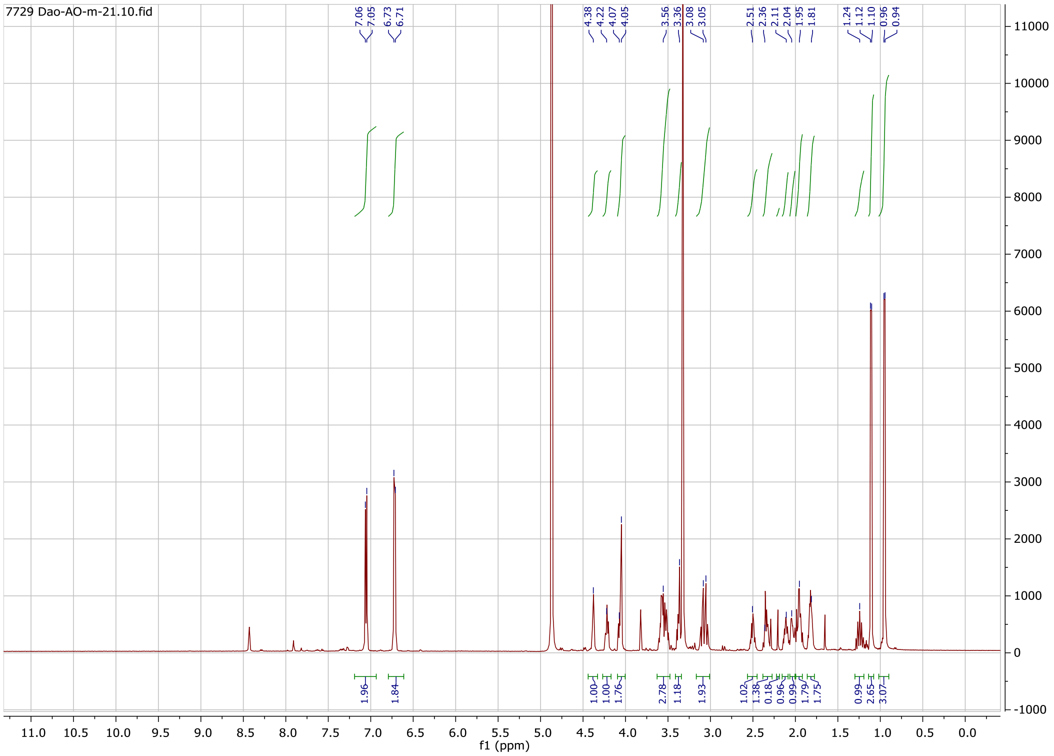

Supplementary Figure 17: ^{1}H NMR spectrum (CD_3OD, 500 MHz) of the mixture **3** and **4**.

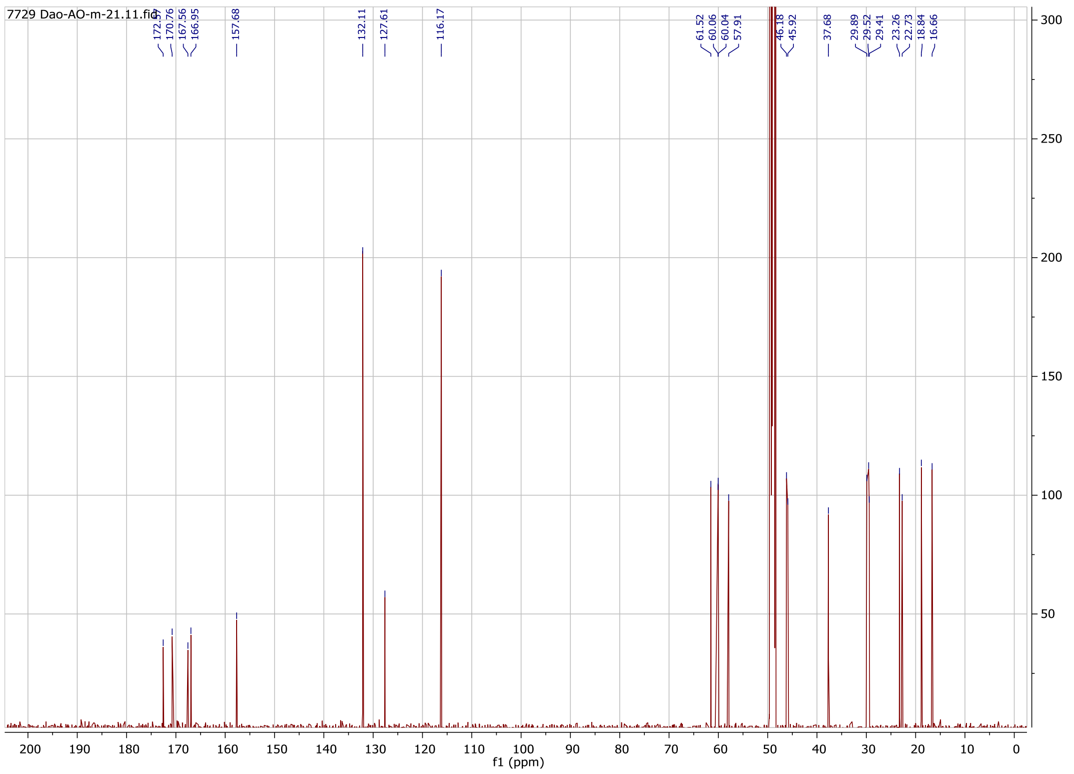

Supplementary Figure 18: ^{13}C NMR spectrum (CD_3OD, 125 MHz) of the mixture **3** and **4**.

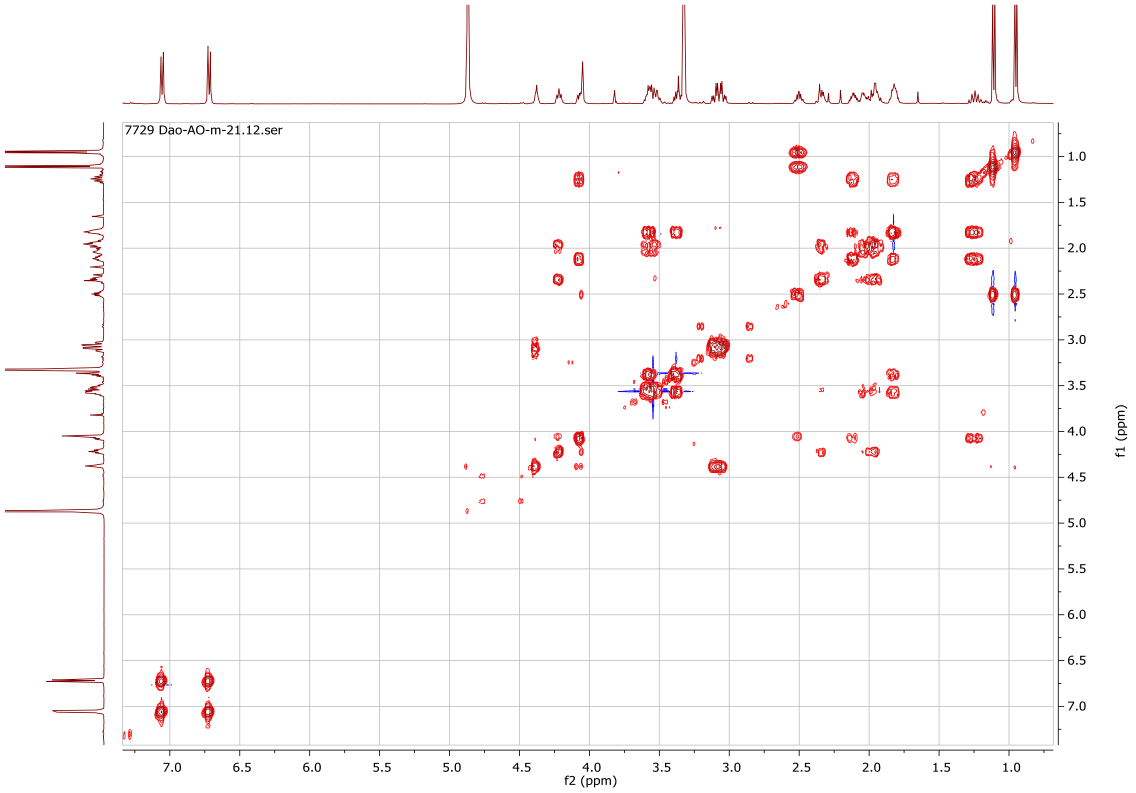

Supplementary Figure 19: COSY spectrum of the mixture **3** and **4**.

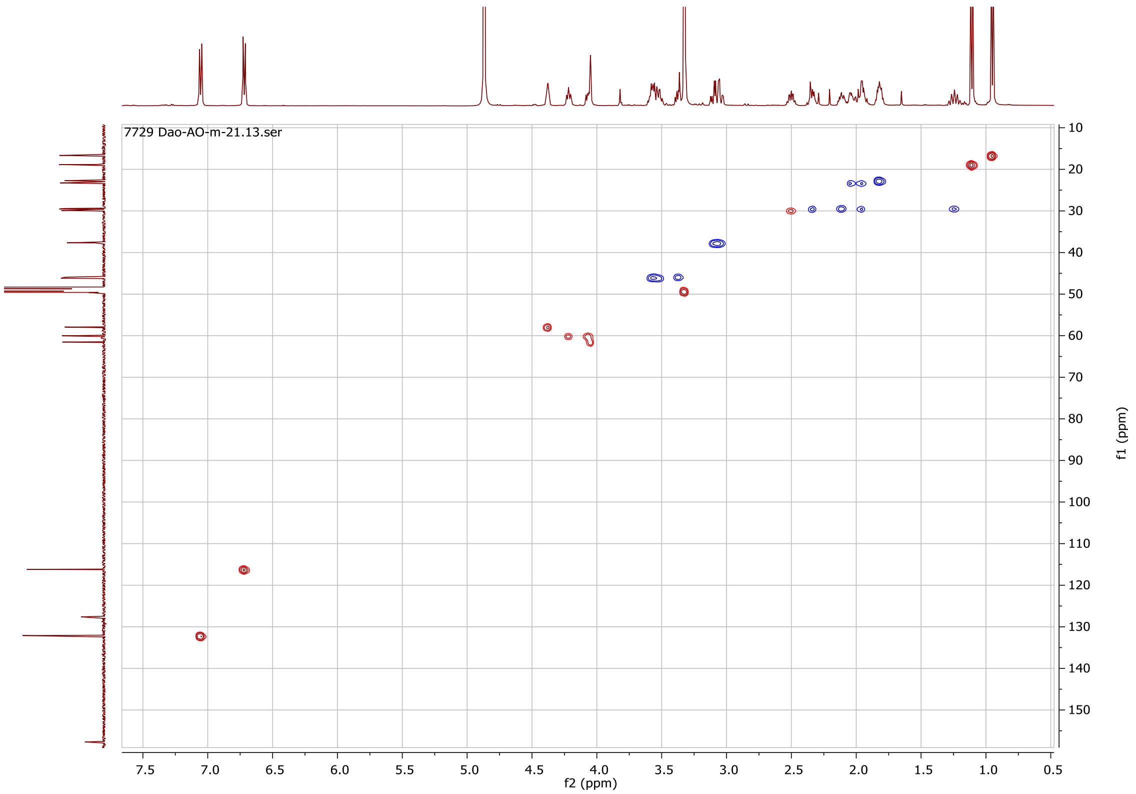

Supplementary Figure 20: HSQC spectrum of the mixture **3** and **4**.

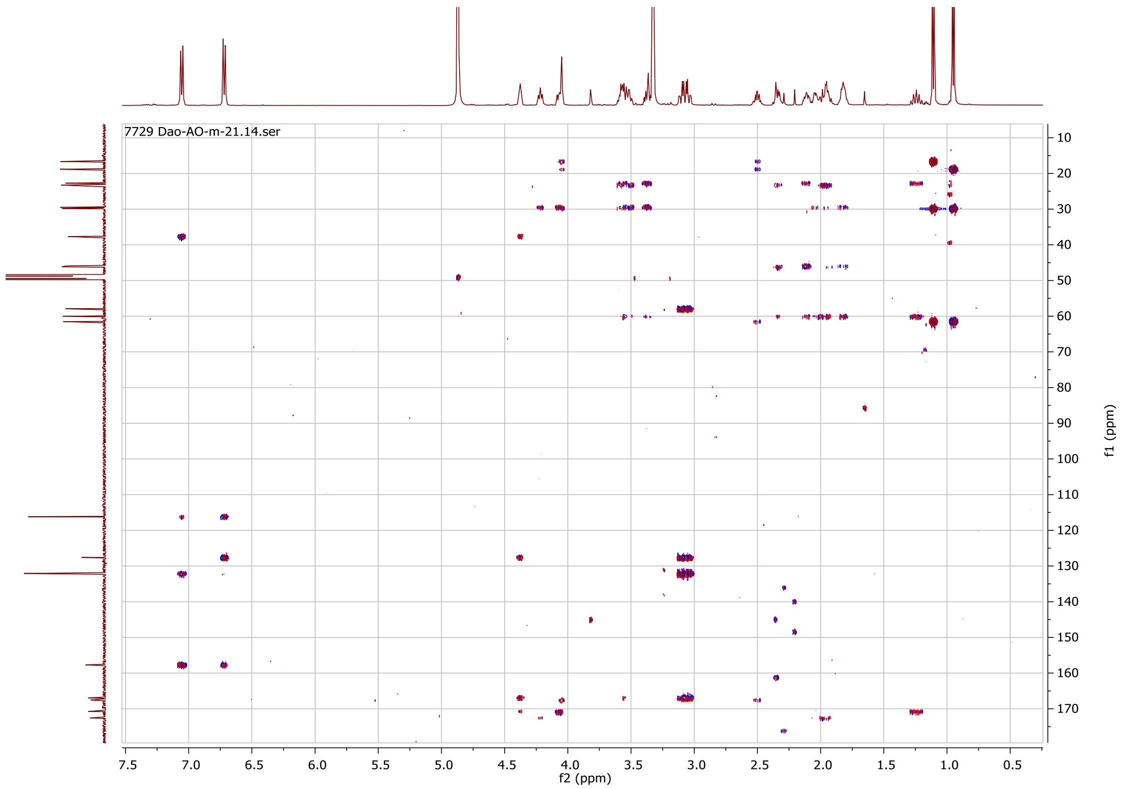

Supplementary Figure 21: HMBC spectrum of the mixture **3** and **4**.

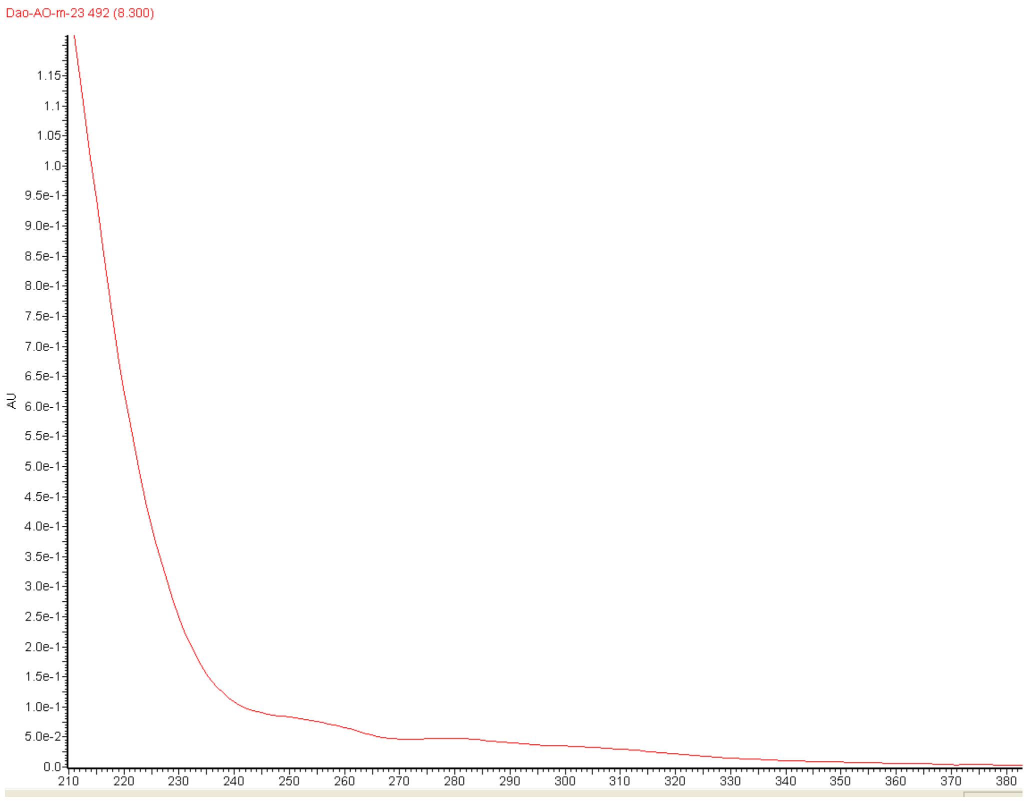

Supplementary Figure 22: UV spectrum of **5**.

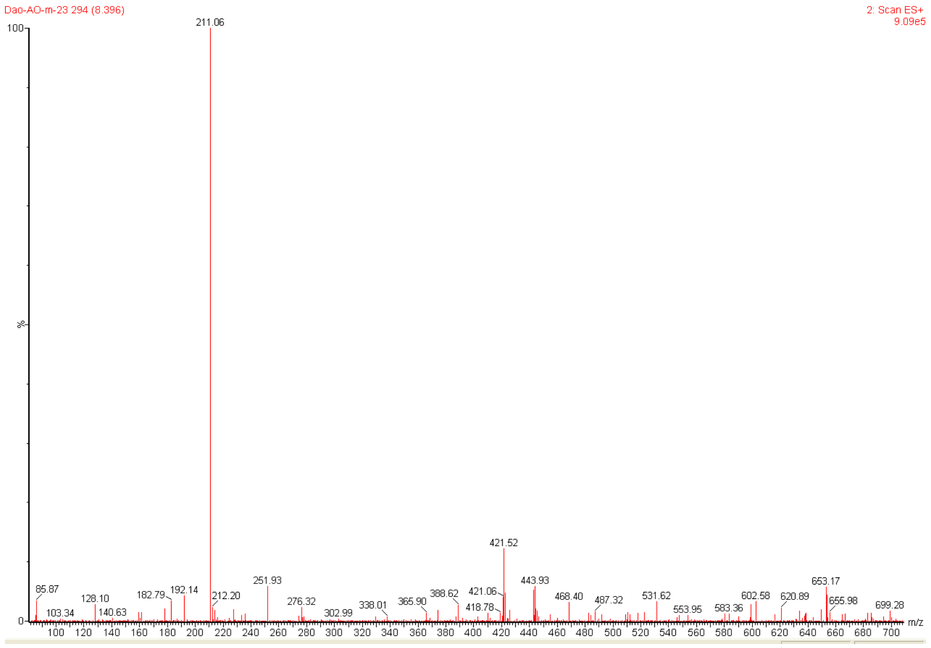

Supplementary Figure 23: Mass spectrum (ES^+) of **5**.

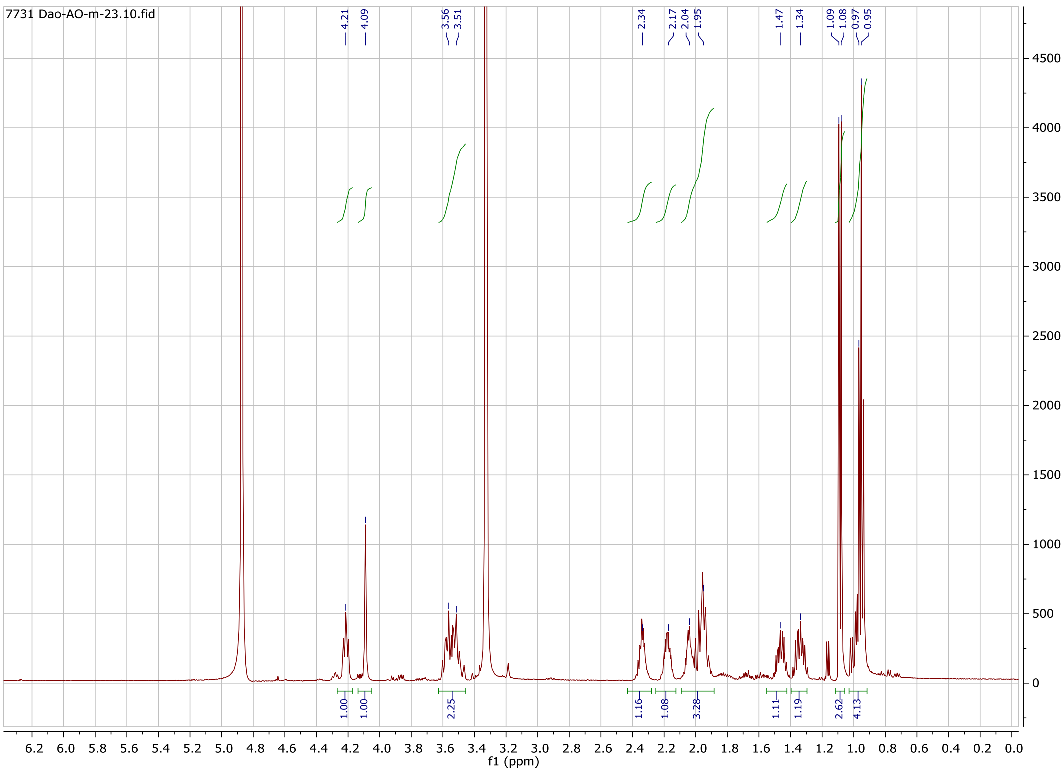

Supplementary Figure 24: ^{1}H NMR spectrum (CD_3OD, 125 MHz) of **5**.

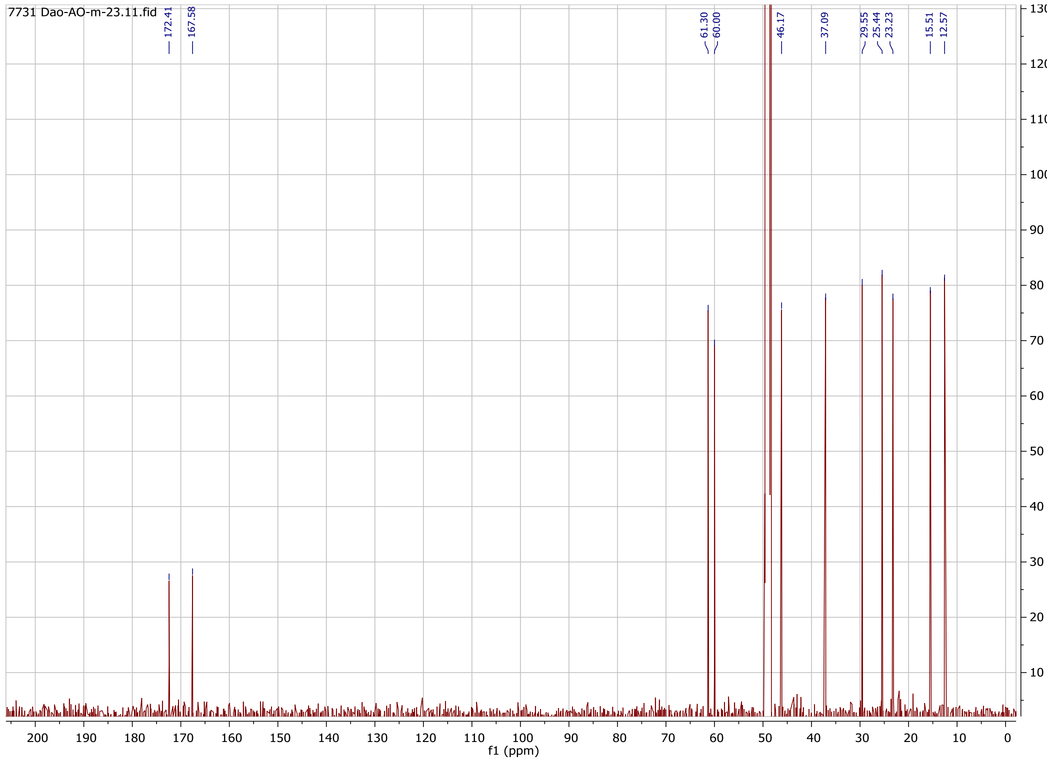

Supplementary Figure 25: ^{13}C NMR spectrum (CD_3OD, 125 MHz) of **5**.

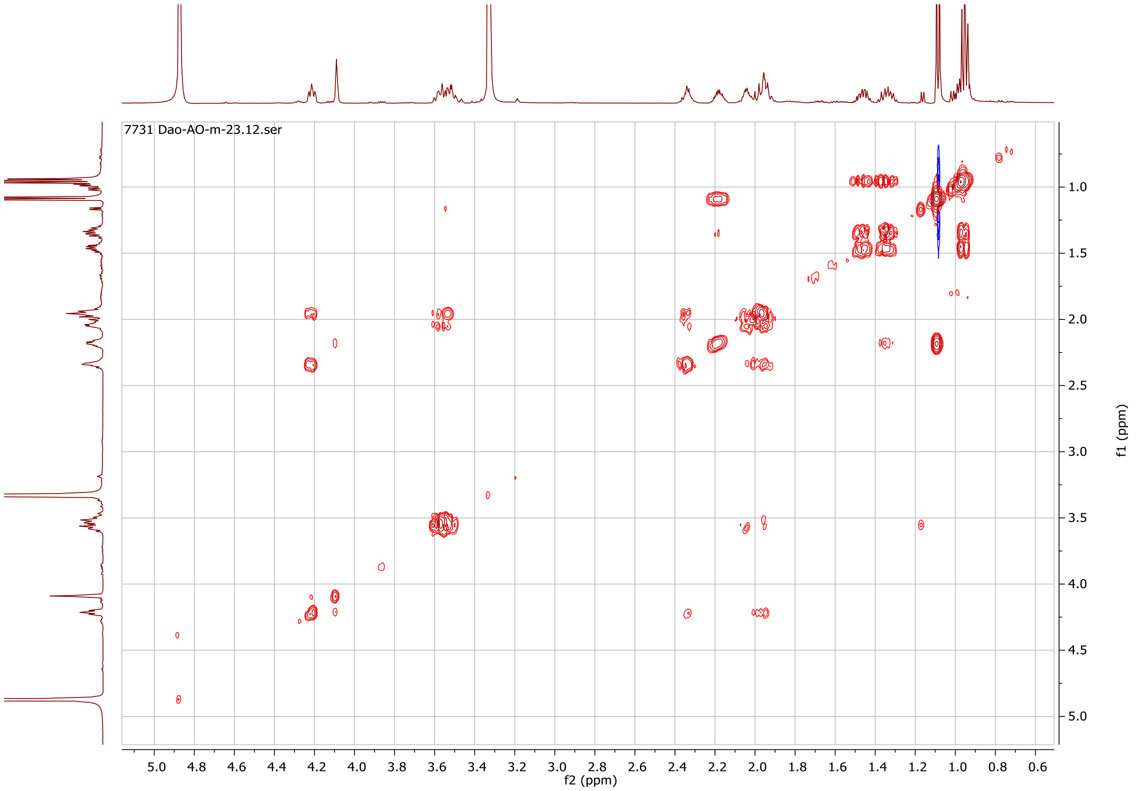

Supplementary Figure 26: COSY spectrum of **5**.

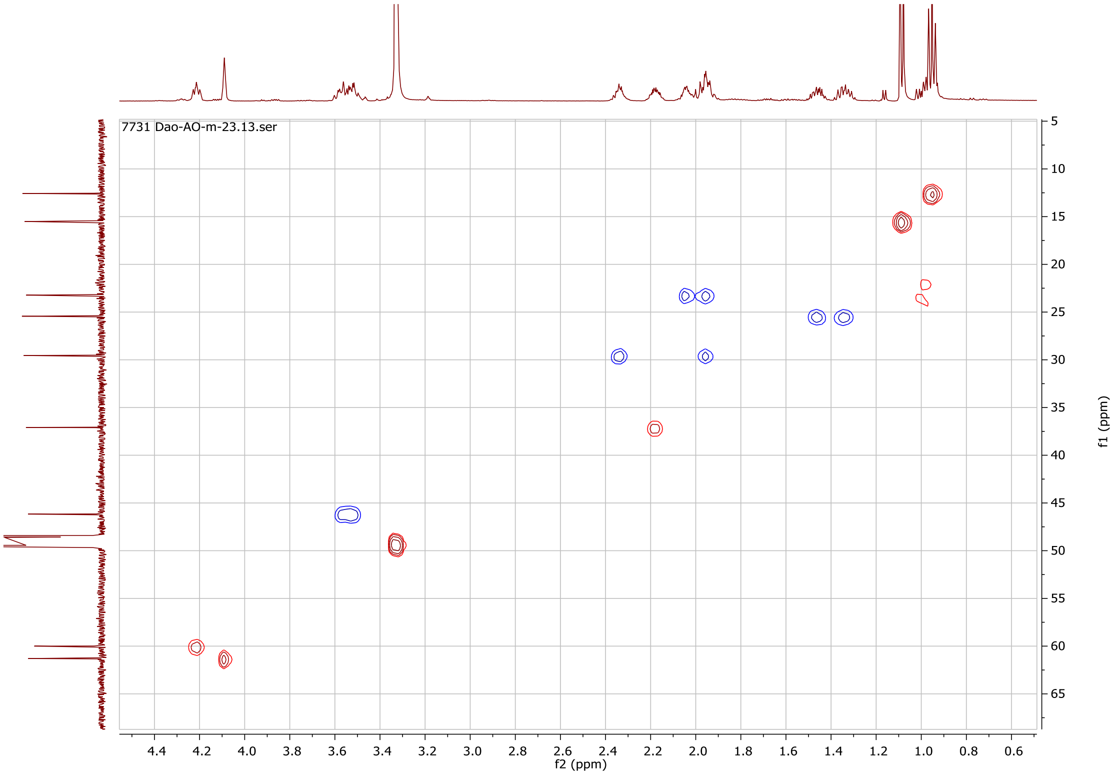

Supplementary Figure 27: HSQC spectrum of **5**.

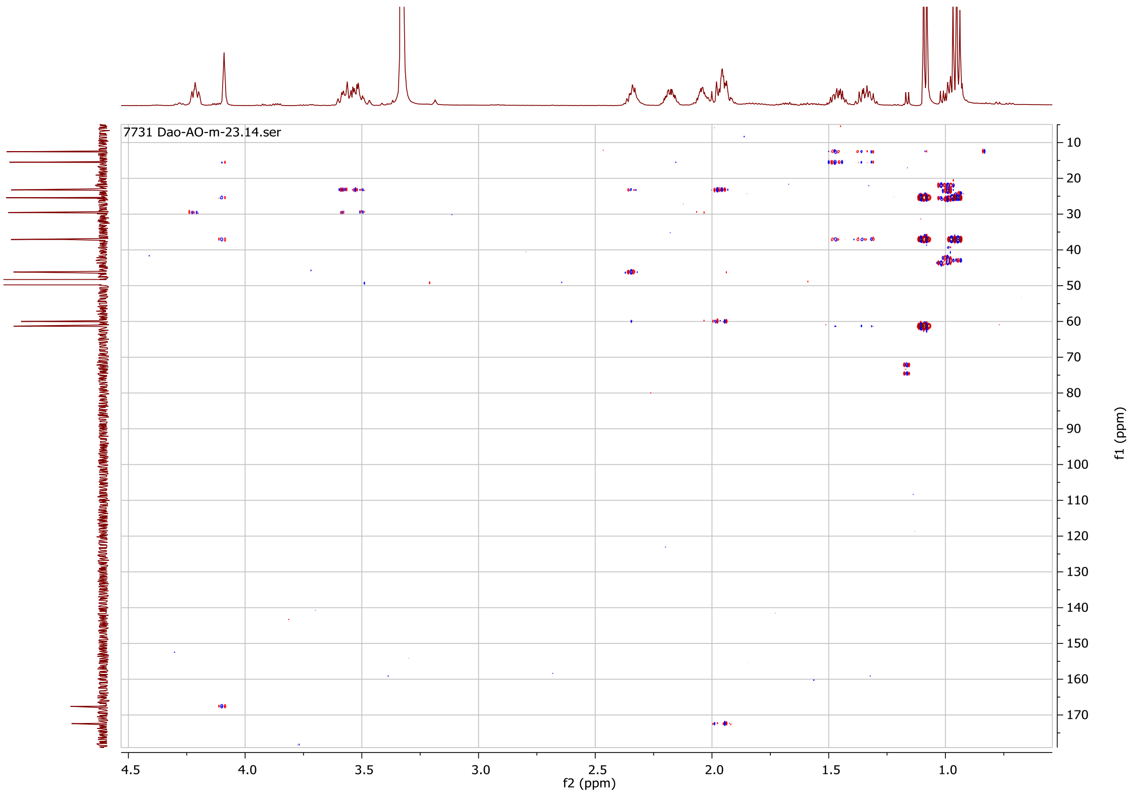

Supplementary Figure 28: HMBC spectrum of **5**.

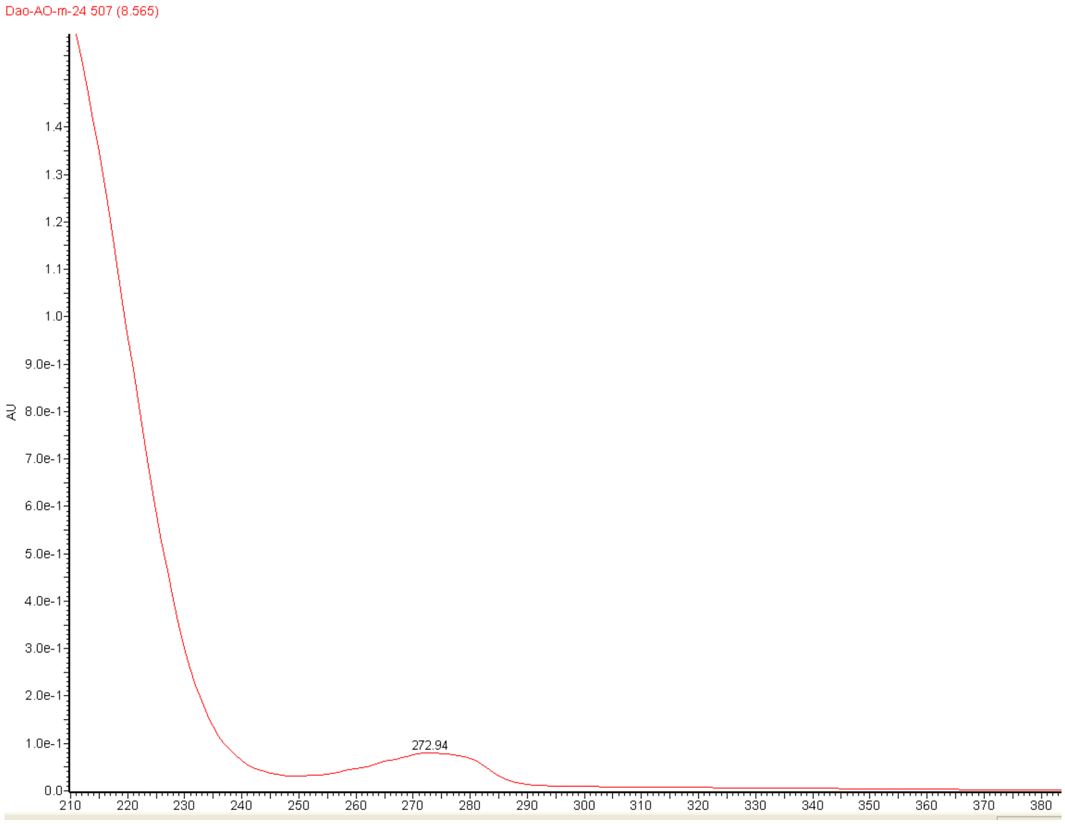

Supplementary Figure 29: UV spectrum of **6**.

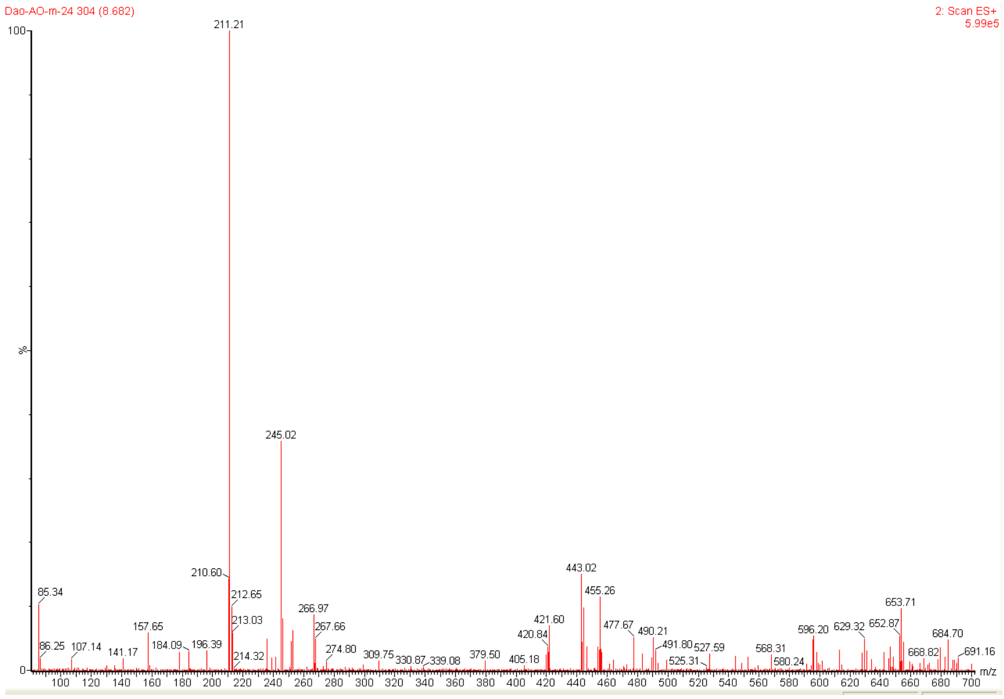

Supplementary Figure 30: Mass spectrum (ES^{+}) of **6**.

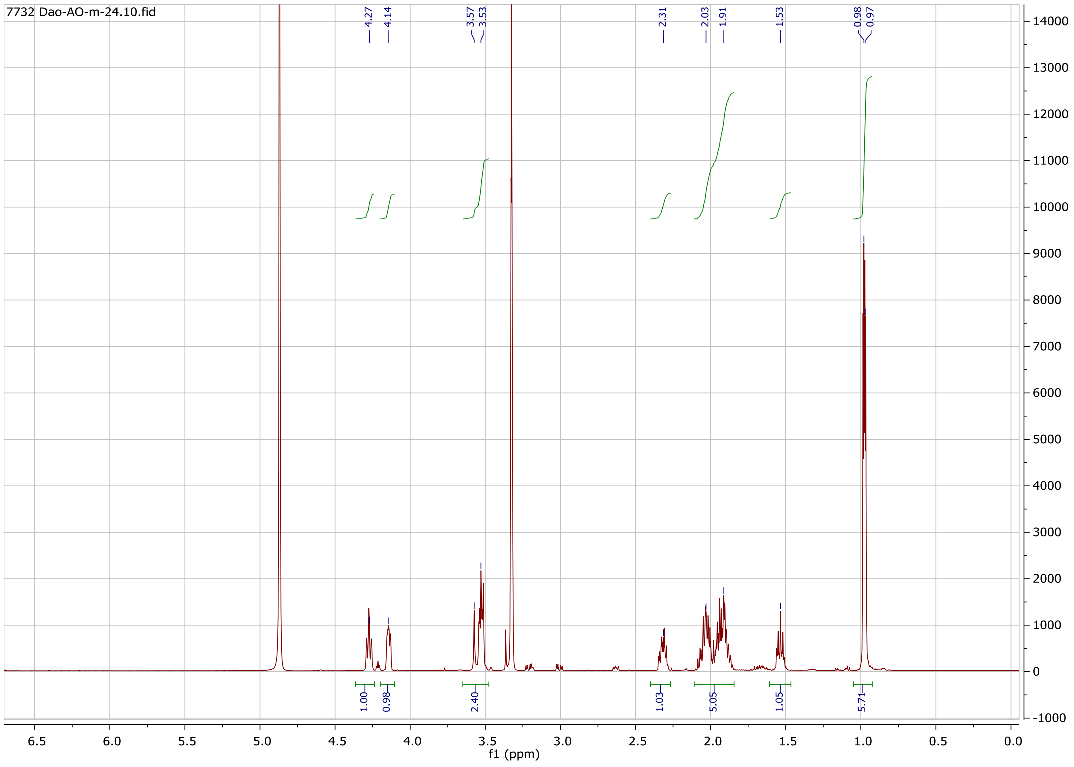

Supplementary Figure 31: ^{1}H NMR spectrum (CD_3OD, 125 MHz) of **6**.

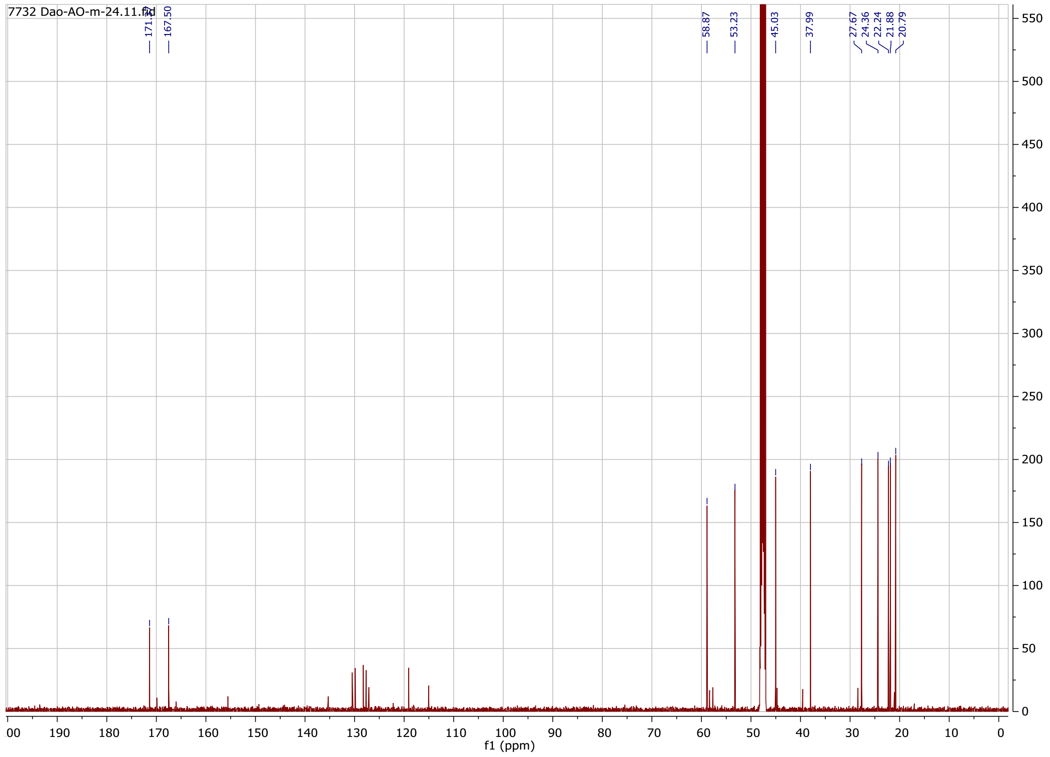

Supplementary Figure 32: ^{13}C NMR spectrum (CD_3OD, 125 MHz) of **6**.

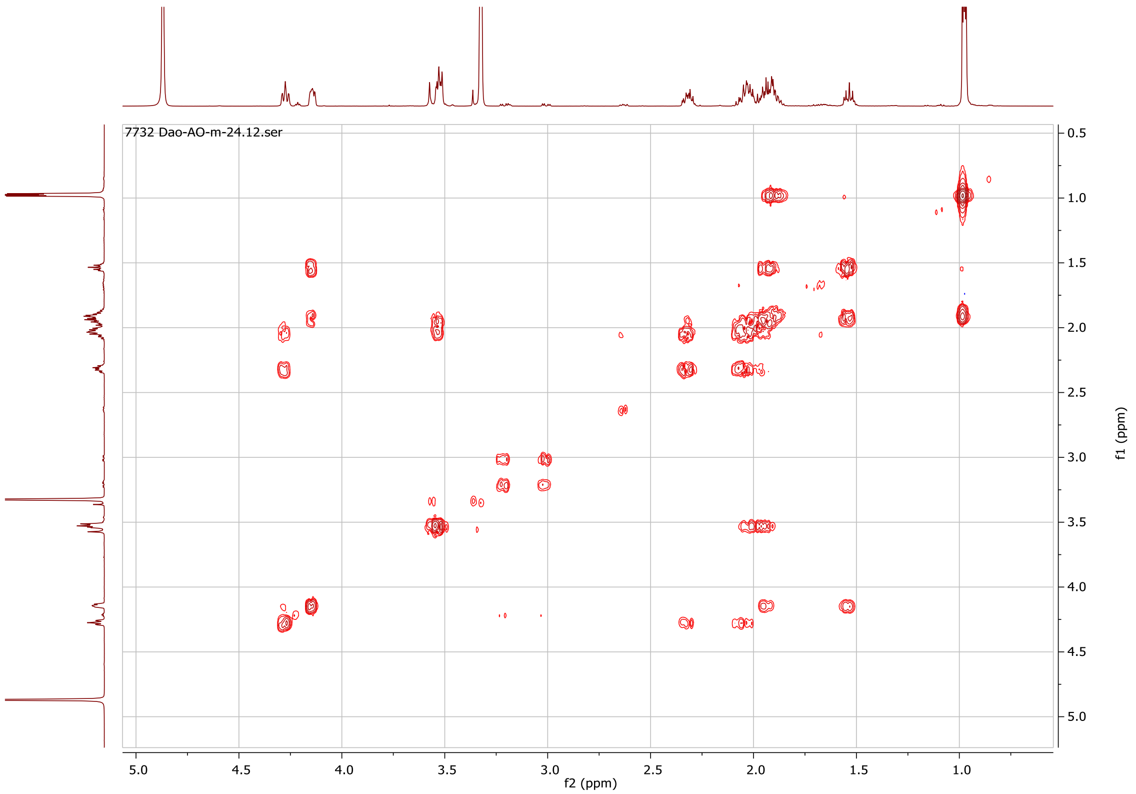

Supplementary Figure 33: COSY spectrum of **6**.

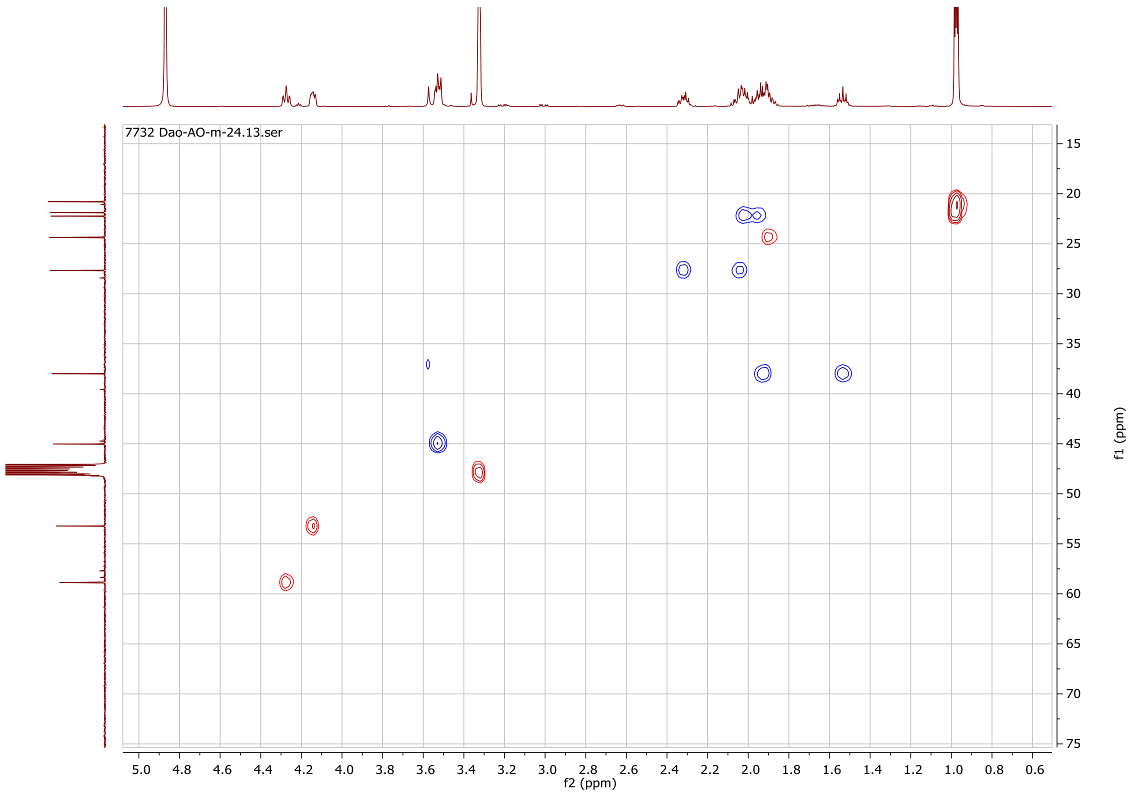

Supplementary Figure 34: HSQC spectrum of **6**.

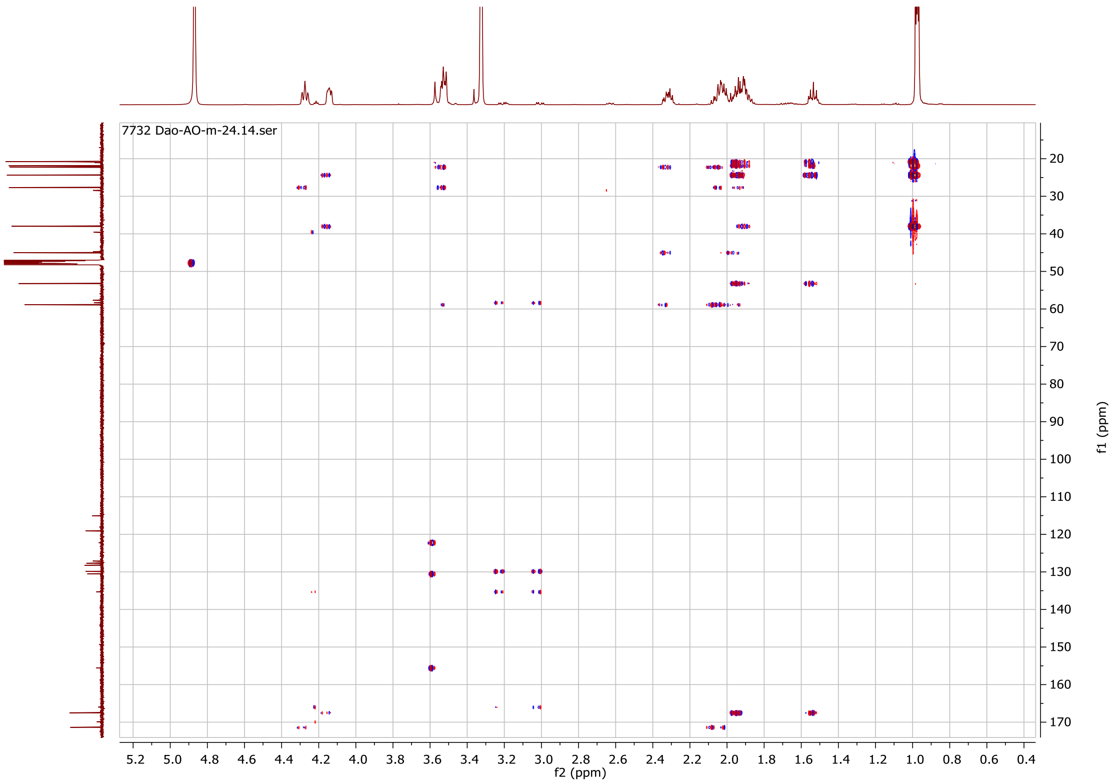

Supplementary Figure 35: HMBC spectrum of **6**.

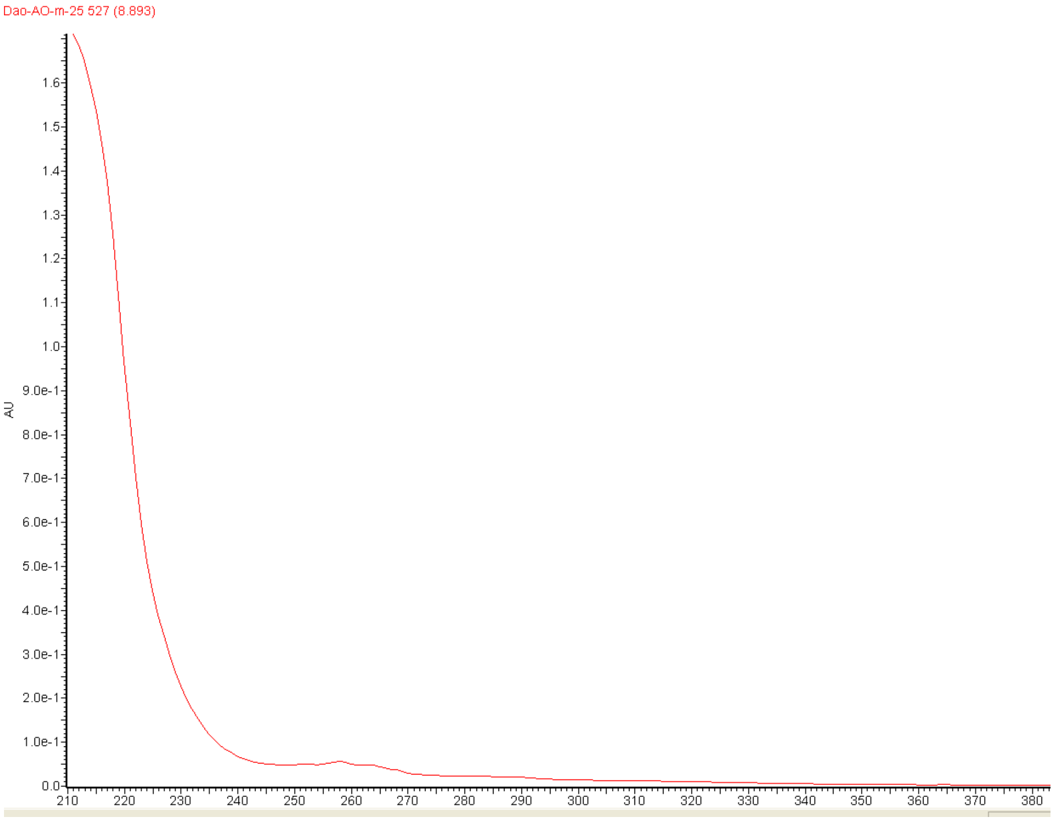

Supplementary Figure 36: UV spectrum of **7**.

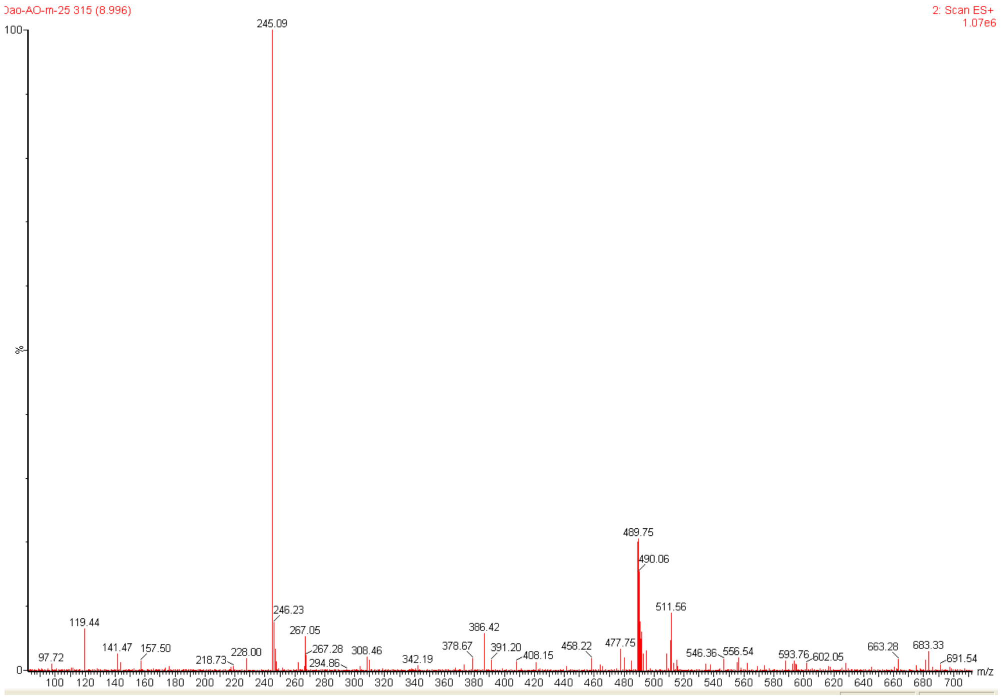

Supplementary Figure 37: Mass spectrum (ES^+) of **7**.

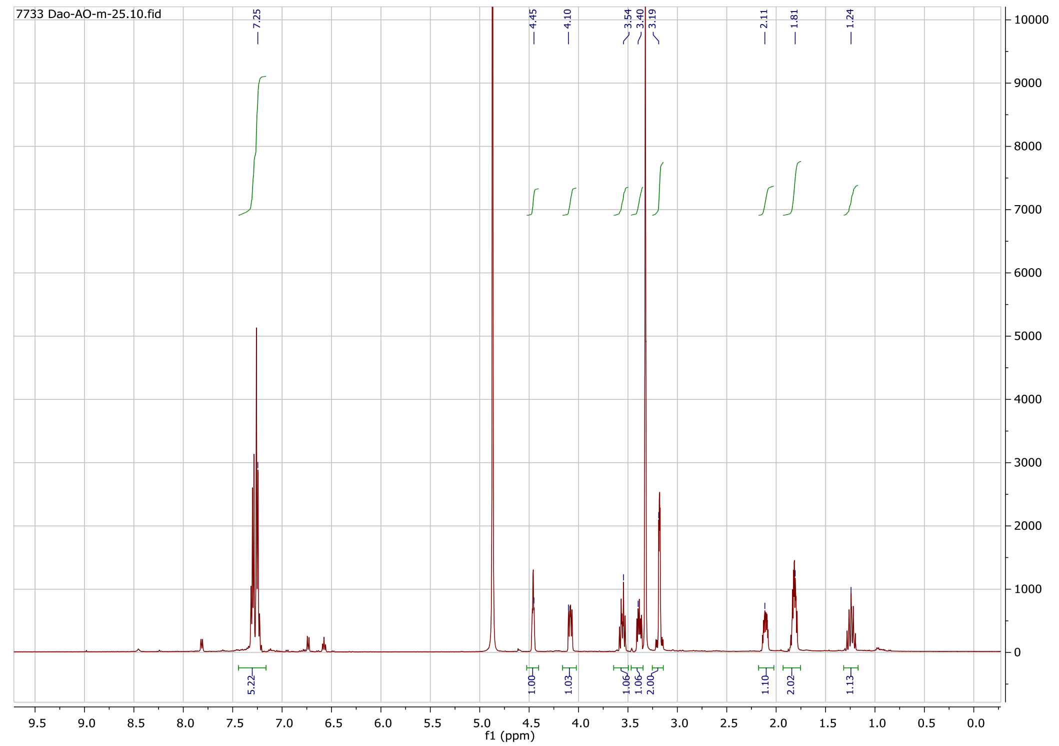

Supplementary Figure 38: ^{1}H NMR spectrum (CD_3OD, 500 MHz) of **7**.

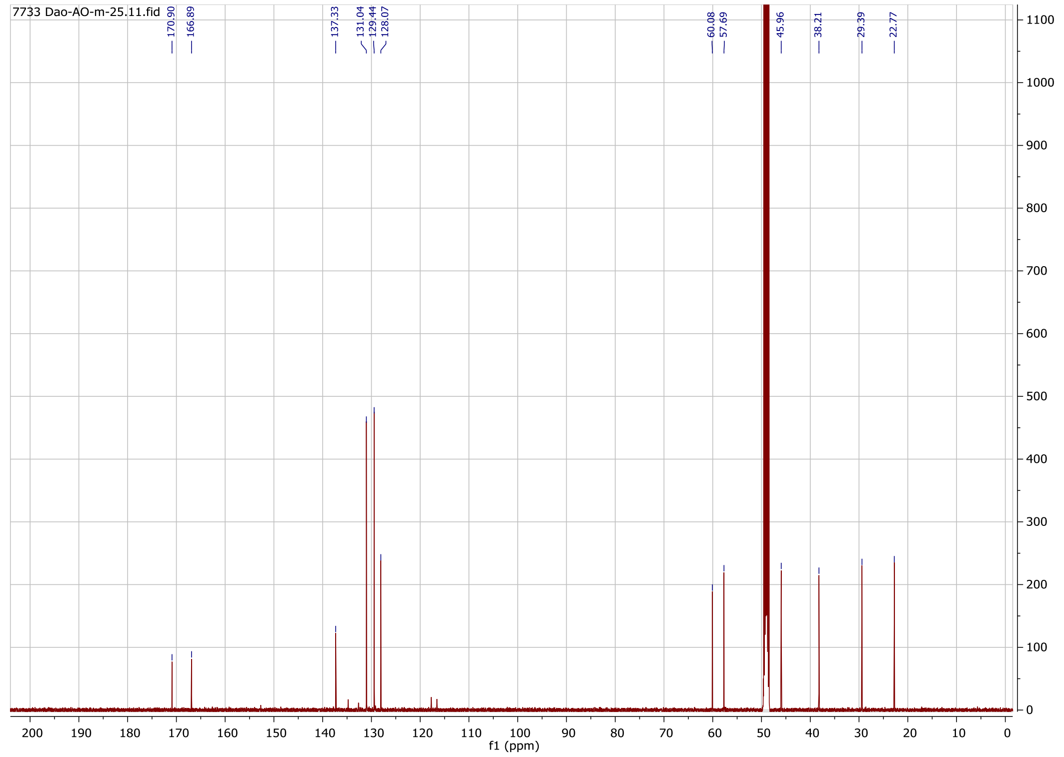

Supplementary Figure 39: ^{13}C NMR spectrum (CD_3OD, 125 MHz) of **7**.

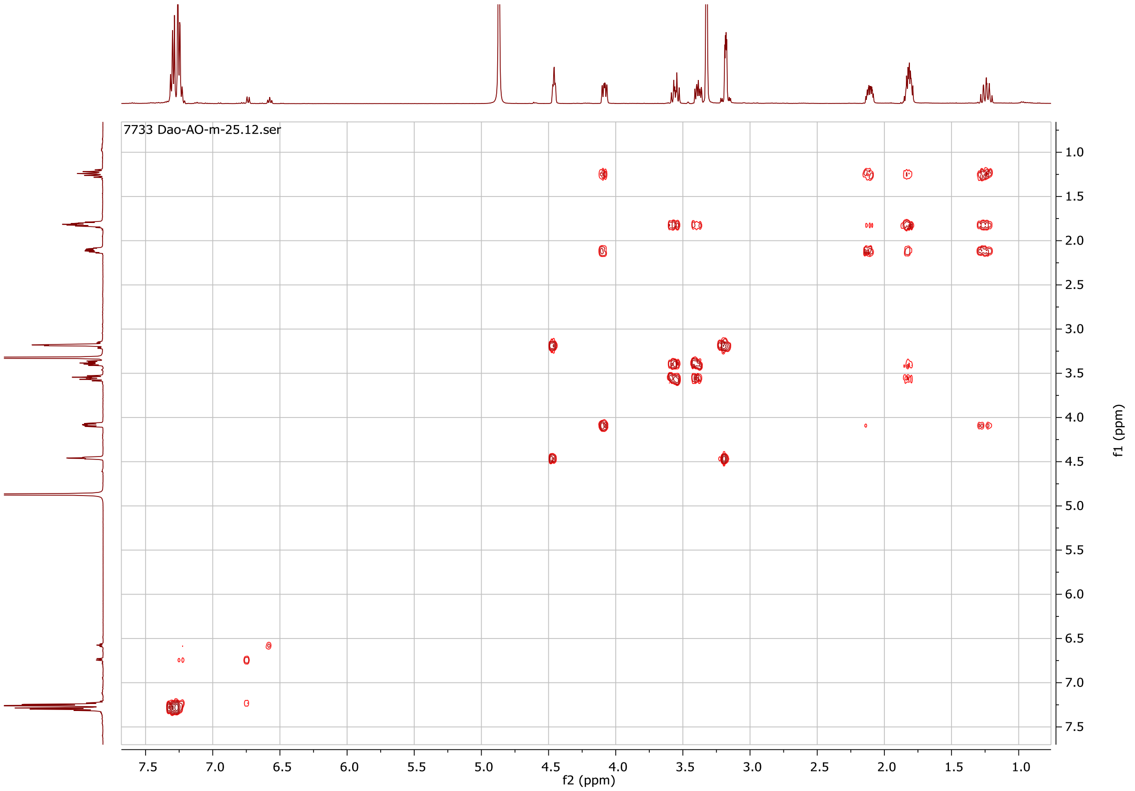

Supplementary Figure 40: COSY spectrum of **7**.

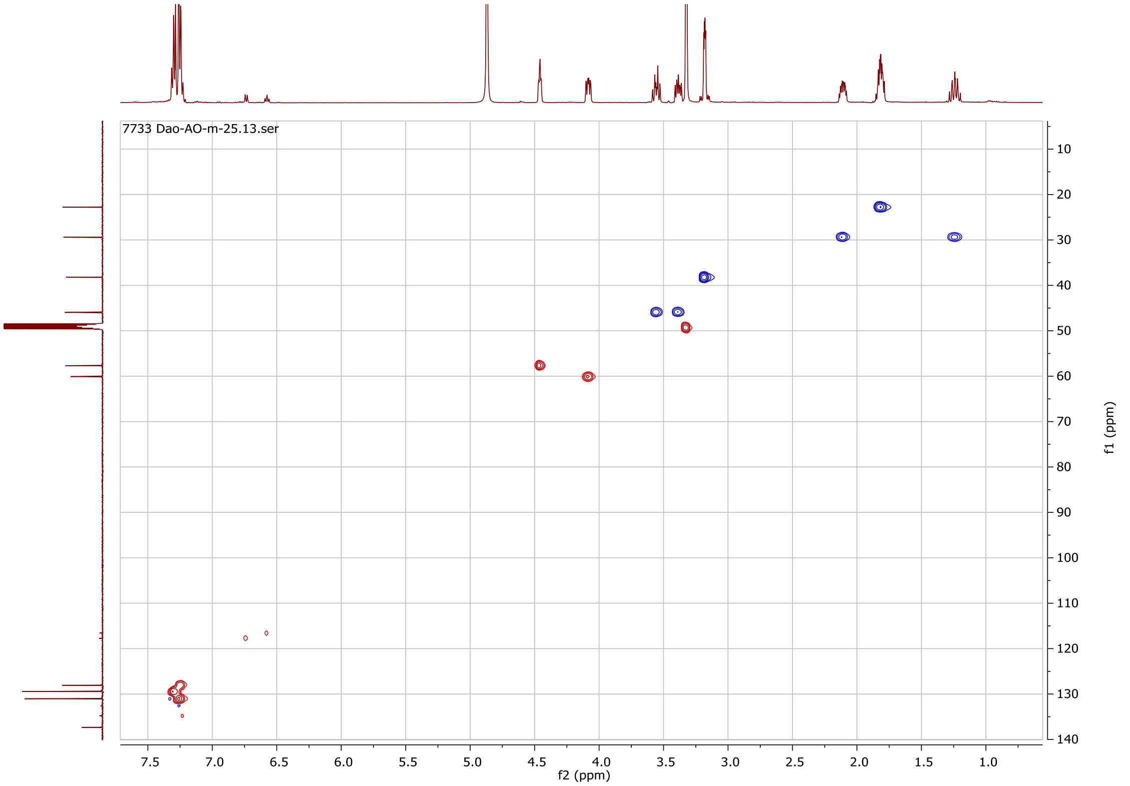

Supplementary Figure 41: HSQC spectrum of **7**.

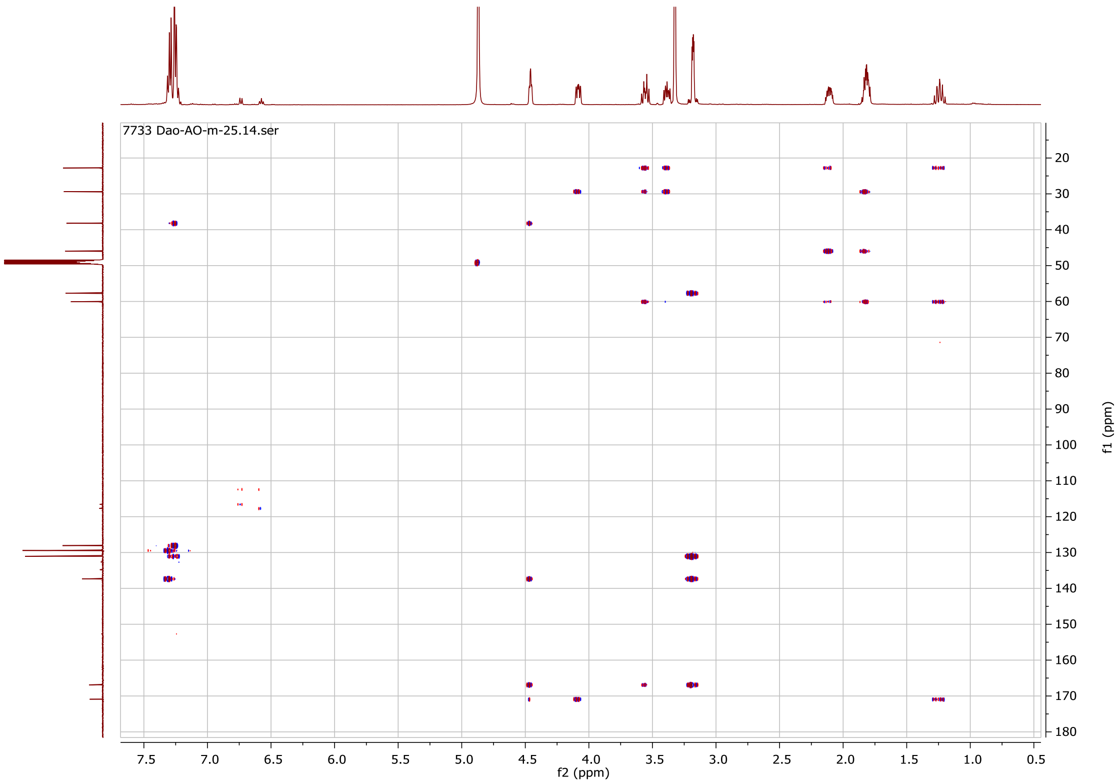

Supplementary Figure 42: HMBC spectrum of **7**.

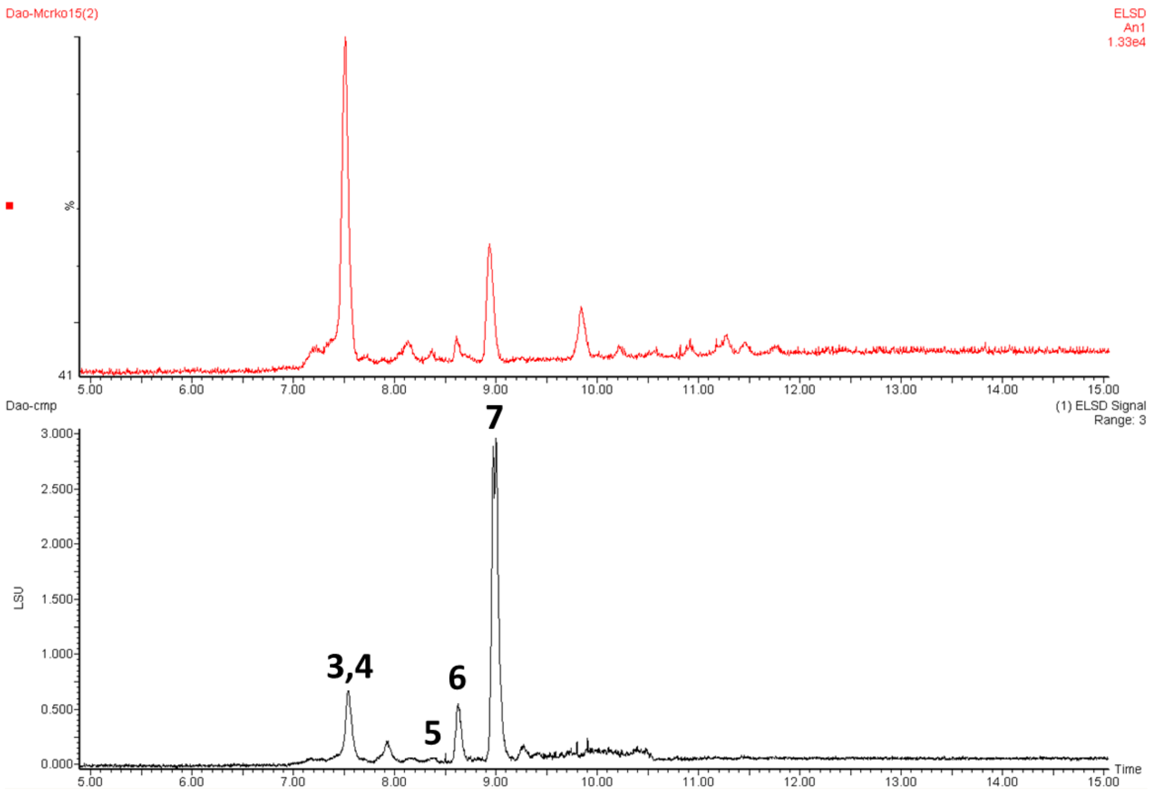

Supplementary Figure 43: LCMS traces (ELSD) comparing the crude extracts of the *mcrA* deletion strain AoΔ*mcrA*-7 (top trace) and CMP media (bottom trace). Isolated diketopiperazines **3**–**7** were shown to be present in the media extract in the absence of *A. oryzae*.

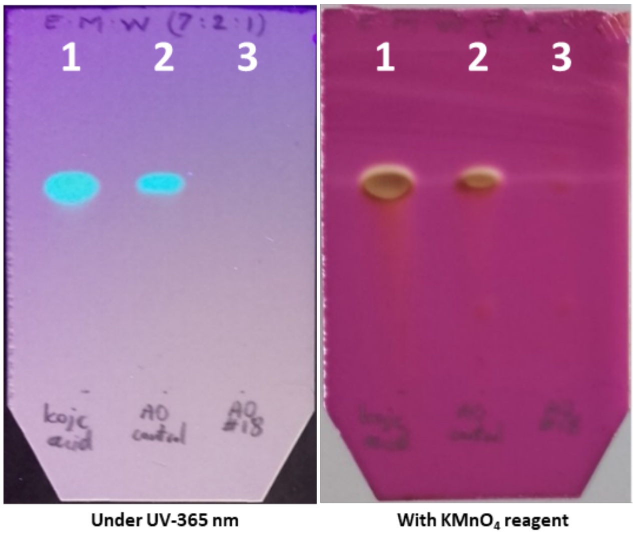

Supplementary Figure 44: TLC analysis showing an absence of kojic acid in the crude extract of *A. oryzae* strain NSARΔK, where gene *kojA* has been disrupted. **1:** Kojic acid standard. **2:** *A. oryzae* NSAR1 parental strain. **3:** Strain NSARΔK.

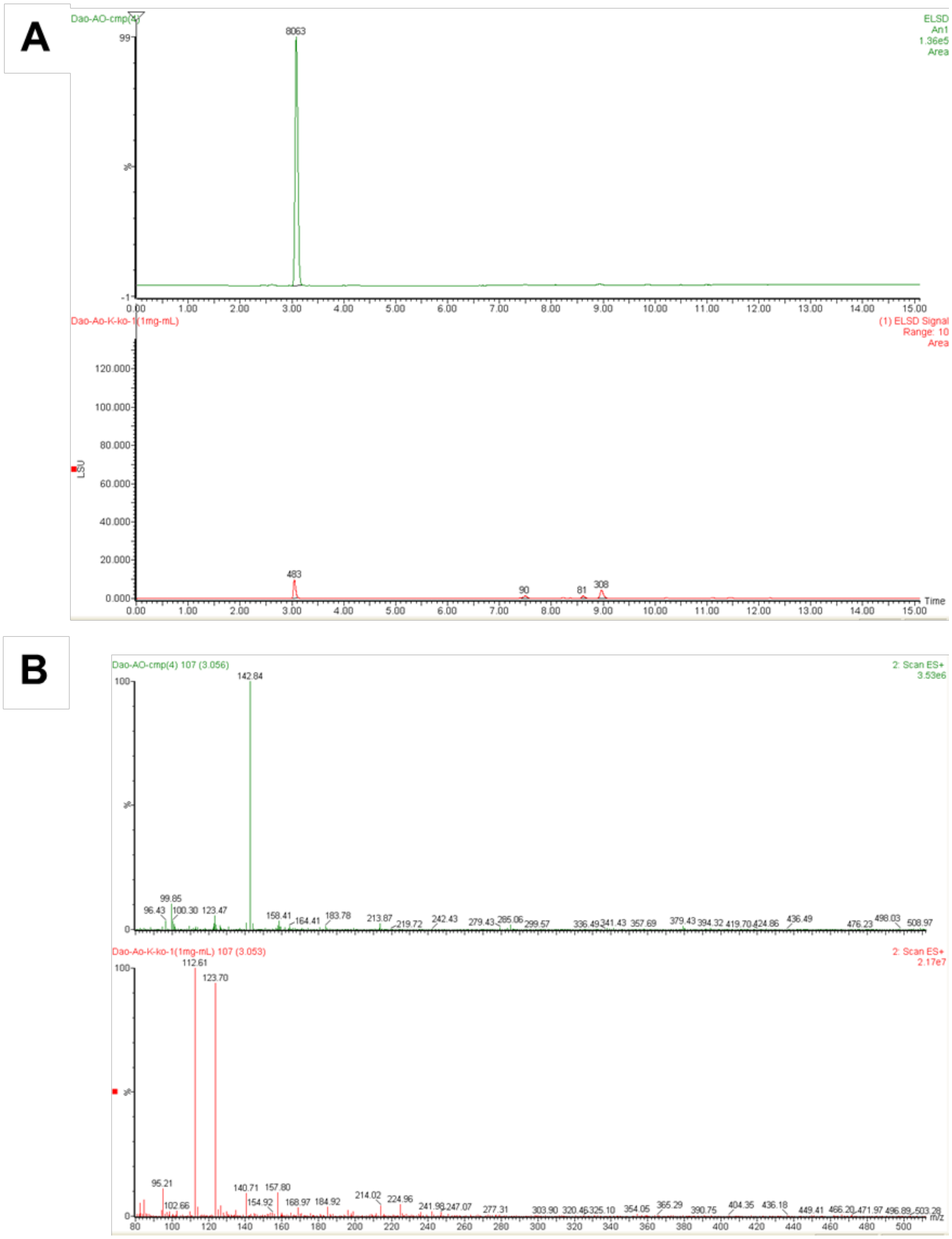

Supplementary Figure 45: HPLC analysis confirming the absence of kojic acid in the crude extract of *A. oryzae* NSARΔK. **(A)** Chromatogram (ELSD) traces of the crude extracts of *A. oryzae* strain NSAR1 (top trace) and the kojic acid knock-out strain NSARΔK. **(B)** Mass spectrum (ES^+) of the peaks at 3.0 min (the retention time for kojic acid) showing that the mass for kojic acid is present in NSAR1 but absent in the knock-out strain NSARΔK.

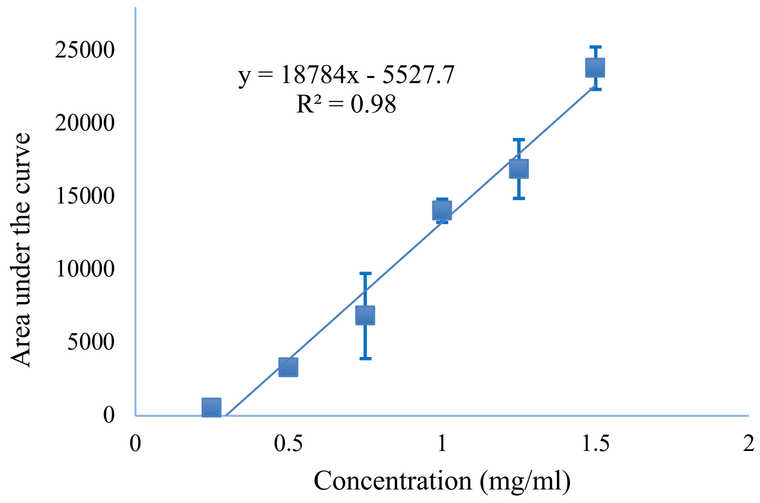

Supplementary Figure 46: Calibration curve for the quantification of kojic acid yields by LCMS. The area under the curve for kojic acid detected using an electron light scattering detector (ELSD) and was shown to have a linear relationship with concentration.

Supplementary Table 1: Quantification of kojic acid production in strain NSAR1 and the transformant *Ao*Δ*mcrA*-7.

	Total extract from 5 ml	Total crude extract per litre	Kojic acid in total extract (%)	Kojic acid yield (g/L)
NSAR1	7.1 ± 2.1 mg	1.42 ± 0.42 g/L	86.5 ± 14.9	1.23 g/L ± 0.21 g/L
*Ao*Δ*mcrA*-7	13.5 ± 3.8 mg	2.7 ± 0.76 g/L	93.4 ± 14.5	2.52 g/L ± 0.39 g/L
